# Cellulose and Nanocellulose Emulsions in Biomedical Applications: From Fundamental Mechanisms to Therapeutic Translation

**DOI:** 10.3390/polym18161986

**Published:** 2026-08-14

**Authors:** Ilker S. Bayer

**Affiliations:** I2Pure Corporation, 44679 Endicott Drive, Suite 300, Ashburn, VA 20147, USA; ilker@i2pure.com

**Keywords:** cellulose nanocrystals, nanocellulose, Pickering emulsion, drug delivery, antimicrobial, bacterial cellulose, vaccine adjuvants, regulatory gaps

## Abstract

Poor aqueous solubility remains one of the most persistent challenges in pharmaceutical development, limiting clinical translation and requiring innovative formulation strategies; approximately 40% of newly discovered pharmaceutical compounds are affected, underscoring the scale of the problem. Emulsion-based delivery systems overcome this limitation by maintaining drugs in a dissolved state, increasing absorption surface area, and enabling controlled release; however, conventional emulsions face thermodynamic instability and coalescence challenges. Cellulose and nanocellulose—cellulose nanocrystals (CNCs), cellulose nanofibers (CNFs), and bacterial cellulose (BC)—have emerged as sustainable, biocompatible alternatives to synthetic surfactants for stabilizing emulsions via Pickering mechanisms involving irreversible adsorption of solid particles at the oil–water interface. This review synthesizes 142 references across eight application themes: fundamentals and history, emulsion templating, drug encapsulation, antimicrobial and pathogen applications, vaccine adjuvants, topical and transdermal delivery, commercial translation, and regulatory gaps. Rather than treating all sources equally, 37 primary studies are examined in depth through structured critical-appraisal tables organized by system type, goal, key result, and limitation; the remainder are synthesized at the pattern level. A key mechanistic distinction is identified between BC as a standalone biomedical material (used in wound dressings, tissue scaffolds, and drug delivery membranes) and BC as a source for Pickering-emulsion stabilizers after disintegration into nanocrystals or nanofibrils. The review’s overall assessment is that the fundamental materials science of cellulose Pickering emulsions is mature and consistent across sources, while the translational evidence, including in vivo confirmation of drug release performance, biofilm-relevant antimicrobial testing, standardized nanocellulose characterization, and up-to-date intellectual property mapping, remains the binding constraint on clinical and commercial adoption. Six specific, evidence-linked research priorities are identified to advance cellulose emulsions toward regulatory approval and clinical use.

## 1. Introduction

Approximately 40% of new chemical entities discovered by pharmaceutical screening programs face commercialization challenges due to poor water solubility, a problem that is particularly acute for lipophilic compounds classified as Biopharmaceutics Classification System (BCS) Class II or IV drugs [1,2]. Simple aqueous solutions are inadequate for these compounds: lipophilic drugs remain undissolved in the gastrointestinal tract, precipitate upon dilution, and exhibit erratic and subtherapeutic oral bioavailability [2]. While conventional approaches—including surfactant solubilization, solid dispersions, and cyclodextrin complexation—have achieved limited success, they introduce their own liabilities: synthetic surfactants can irritate mucous membranes, solid dispersions may lack robust stability, and high excipient burdens complicate regulatory pathways [1].

Emulsion-based drug delivery systems address these shortcomings by dissolving lipophilic drugs in an oil phase and emulsifying with a stabilizing agent. Emulsions maintain drugs in a dissolved state, avoiding the rate-limiting dissolution step; dramatically increase the interfacial area available for absorption; protect chemically labile drugs from degradation; enable lymphatic transport that bypasses first-pass hepatic metabolism for highly lipophilic compounds; and permit fine control over release kinetics [1,2,3]. Yet emulsions introduce a thermodynamic trade-off: they are inherently unstable systems susceptible to coalescence, Ostwald ripening, and flocculation—destabilization mechanisms that degrade efficacy, reduce shelf life, and complicate manufacturing scale-up [4,5].

Cellulose and its nanostructured derivatives—cellulose nanocrystals (CNCs), cellulose nanofibers (CNFs), and bacterial cellulose (BC)—have emerged as biocompatible, sustainable alternatives to synthetic surfactants, stabilizing emulsions via Pickering mechanisms in which solid colloidal particles irreversibly adsorb at the oil–water interface and physically prevent coalescence without sacrificing drug solubility or biocompatibility [6,7,8]. This review accordingly emphasizes nanocellulose, since it currently dominates the Pickering-emulsion biomedical literature; conventional (non-nano) cellulose derivatives such as methylcellulose, hydroxypropyl methylcellulose, and carboxymethylcellulose appear here mainly as rheology modifiers, viscosity-building excipients, or co-stabilizers rather than as primary emulsion stabilizers in their own right.

Over the past two decades, cellulose-stabilized emulsions have demonstrated versatility across biomedical applications: oral and transdermal drug delivery, antimicrobial therapeutics, vaccine adjuvants, tissue-engineering scaffolds, and topical/cosmetic formulations [9,10,11]. Despite this breadth of evidence, cellulose emulsions remain underutilized in clinical practice, and regulatory frameworks remain uncertain regarding characterization standards, safety data, and excipient classification—gaps this review addresses.

### Literature Search Strategy

An initial pool of over 200 candidate sources was screened for relevance, duplication, and methodological reporting quality, yielding the 142 references retained in this review (see Section 9). Rather than treating these 142 sources as equivalent, this review presents a smaller set of methodologically pivotal studies in narrative form, chosen because they resolve an open question, expose an inconsistency, or correct a common misattribution. The remaining primary literature is presented in structured critical-appraisal tables (system type, goal, design, key result, limitation) rather than repeated prose—keeping verified detail visible while allowing direct comparison across studies.

Research was identified through iterative keyword searches of Web of Science and Google Scholar, covering primarily 2011–2025 (the most recent 15 years); a small number of older foundational references (11 of 142, dating to 1998) were also included for historical and mechanistic context. Search terms combined “cellulose,” “nanocellulose,” “cellulose nanocrystal,” “nanofibril,” “bacterial cellulose,” and “Pickering emulsion” with application-specific terms such as “drug delivery,” “antimicrobial,” “vaccine adjuvant,” “wound healing,” and “tissue scaffold.” Sources were screened for direct relevance to cellulose- or nanocellulose-stabilized emulsions in a biomedical context, and duplicate, non-English-language, and insufficiently reported studies were excluded, yielding the 142 references retained here. Of these, 37 were selected as primary or anchor studies for in-depth narrative treatment based on methodological completeness and evidentiary weight; the remainder are synthesized at the pattern level within the structured critical-appraisal tables.

This search-and-appraisal approach carries some limitations. Screening relied on keyword-based database searches conducted by [a single reviewer/the author team—confirm as applicable] rather than a formally registered systematic protocol, and no independent second-reviewer or inter-rater agreement check was performed; the search was also limited to English-language publications, which may exclude relevant non-English primary research. The structured critical-appraisal criteria used throughout (system type, goal, design, key result, limitation) reflect an author-defined framework rather than a validated external instrument (e.g., AMSTAR, ROBIS), and publication bias was not formally assessed—given that most primary studies report favorable outcomes, negative or null results may be underrepresented in the literature synthesized here. Because the majority of the 142 references are synthesized at the pattern level rather than individually appraised, some study-level nuance is necessarily condensed; readers requiring study-specific detail should consult the primary sources directly via the reference list.

**Figure 1:**
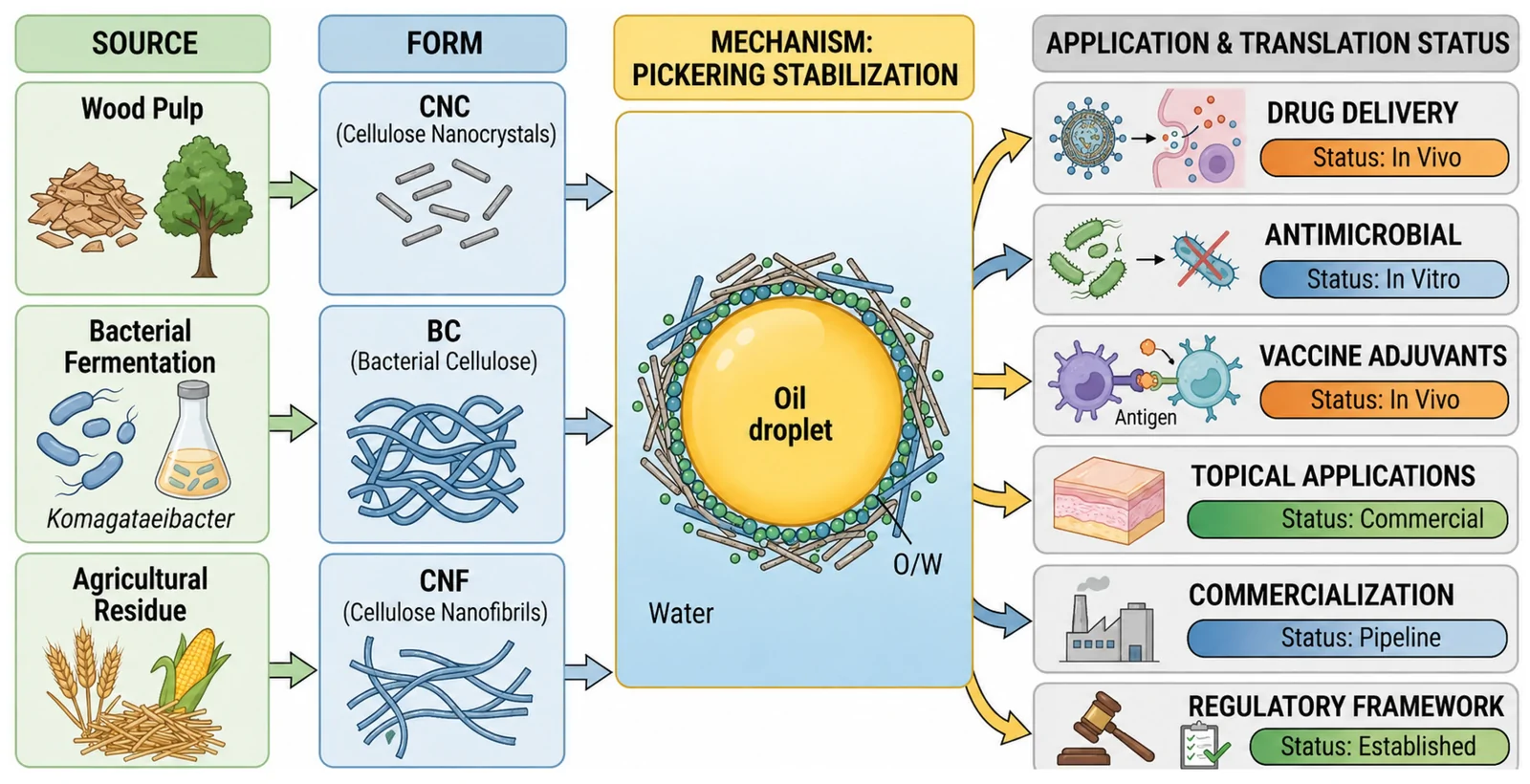
Cellulose emulsions: from source material to clinical translation. The figure maps the three primary cellulose sources—wood pulp (CNC, CNF), agricultural residues (CNF), and bacterial fermentation (BC)—to the Pickering-emulsion stabilization route, the eight biomedical application themes reviewed, and the translational barriers that govern clinical adoption. Note that bacterial cellulose is positioned principally as a standalone biomedical material rather than as a primary Pickering-emulsion stabilizer, as discussed in Section 2.6.

This review is organized into eight thematic sections: (1) Fundamentals and History; (2) Emulsions as Templates; (3) Drug Encapsulation and Formulation; (4) Antimicrobial and Pathogen Applications; (5) Vaccine and Immunotherapeutic Delivery; (6) Topical and Transdermal Delivery; (7) Commercialized Products and Clinical Translation; and (8) Regulatory Gaps and Shortcomings, followed by concluding remarks and research priorities. Figure 1 summarizes this source-to-translation pipeline graphically.

The figure connects three cellulose feedstocks—wood pulp (CNC, CNF), agricultural residues (CNF), and bacterial fermentation (BC)—to the Pickering-emulsion stabilization mechanism, the eight application themes reviewed, and the key barriers that currently separate laboratory performance from clinical translation.

## 2. Fundamentals and History

### 2.1. From Cellulose Ethers to Pickering Nanocellulose

The use of cellulose in emulsion science spans two distinct eras. In the first, cellulose ethers such as methyl cellulose, hydroxypropyl methyl cellulose, and carboxymethyl cellulose served primarily as viscosity enhancers and secondary stabilizers [12,13,14]. In the second, beginning around 2007–2012, cellulose nanocrystals and nanofibers emerged as primary Pickering stabilizers capable of forming exceptionally stable oil-in-water and water-in-oil emulsions [15,16,17]. Ethyl cellulose was recognized early as an effective emulsion stabilizer in its own right [18,19], and comprehensive reviews now document the breadth of nanocellulose-stabilized Pickering systems [6,7,8,20]. Two of those reviews are foundational enough to be examined directly: together they establish the field’s central claim—that particle stabilization is mechanistically superior to surfactant stabilization—while also exposing its central limitation, that the claim rests on pooling results across chemically very different particle systems rather than controlled, particle-matched comparisons (see Table 1 for a detailed comparison of system types, design strategies, and key results across foundations, manufacturing, and engineering applications).

Zoppe et al. [17] and Bai et al. [22] are both primary, well-instrumented mechanistic studies, but both use model oils (heptane, sunflower oil, paraffinic alkanes) rather than pharmaceutical actives, and neither reports multi-month stability under physiological conditions (37 °C, physiological ionic strength, presence of proteins or bile salts). The gap between ‘stable for six months on a benchtop’ and ‘stable in a manufactured drug product under ICH storage conditions’ has not yet been systematically closed for cellulose Pickering emulsions.

### 2.2. Types of Emulsions Stabilized by Cellulose

Cellulose and nanocellulose stabilize a spectrum of emulsion architectures, summarized in Table 2. These range from conventional oil-in-water (O/W) systems for oral and topical delivery [6,29], to water-in-oil (W/O) systems using hydrophobized cellulose [15], and high internal phase emulsions (HIPEs) that serve as templates for porous materials [27,30].

### 2.3. Pickering Stabilization Mechanism

**Figure 2:**
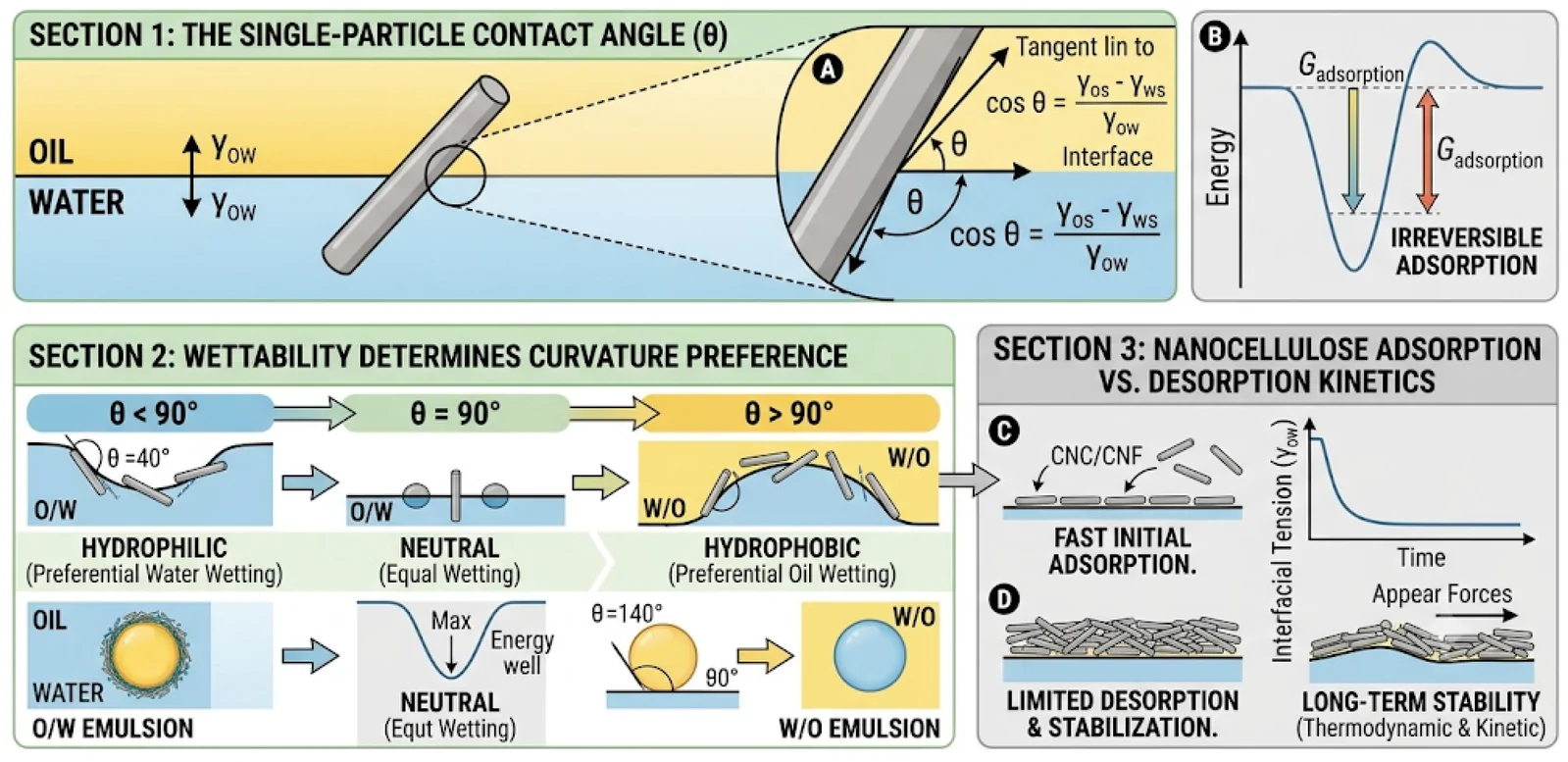
Pickering stabilization mechanism at the oil–water interface. (**A**) Young’s equation in contact angles on the particle surface-liquid interface. (**B**) Changes in the free energy of adsorption across the interface. (**C**) Rapid initial nanocellulose adsorption across the interface and (**D**) Stabilization of the interface with further adsorption and limited desorption of the nanocellulose particles along the interface.

In a Pickering emulsion, solid particles adsorb at the oil–water interface and form a mechanical barrier that prevents droplet coalescence [8,21]. For cellulose nanocrystals, the semicrystalline rod-like particles position themselves so that hydrophobic crystal planes contact the oil phase while hydroxyl-rich planes contact water, producing a densely packed interfacial monolayer [4,16,39]. Because the free energy of desorption for a nanoparticle is many orders of magnitude larger than thermal energy, adsorption is effectively irreversible, conferring stability over months to years [4,6]. Figure 2 illustrates this interfacial geometry schematically.

### 2.4. Sources and Forms of Nanocellulose

A central message of this review is that the Pickering mechanism is essentially universal across cellulose sources, so the choice of feedstock is governed by cost, supply chain, and sustainability rather than fundamental performance. Nanocellulose is produced from wood pulp [34,35], agricultural and fruit residues [42], and bacterial fermentation [26,37]. Table 2 summarizes the principal forms; Reid et al. [23] specifically warrants closer reading because its finding—that commercial CNC batches vary by supplier and hydrolysis method—is a reproducibility risk for the entire downstream literature discussed in Section 4 and Section 5.

### 2.5. Rheology, Stability, and Emulsion-Templated Materials

Cellulose-stabilized emulsions typically display solid-like interfacial rheology, elevated viscosity, and shear-thinning behavior that together enhance resistance to creaming and coalescence [4,43,44]. One study demonstrates that emulsion templating is not just a delivery-vehicle concept but a structural design tool in its own right: converting an open-pore cellulose aerogel into a quasi-closed-pore structure via Pickering-emulsion templating solved a real, previously unresolved thermal-conductivity problem, achieving thermal conductivity as low as 15.5 mW/(m·K) with substantially reduced density [25].

This is a materials-engineering (thermal insulation)—not a biomedical—application, but the same templating logic underlies the tissue-scaffold work discussed in Section 3.

### 2.6. Bacterial Cellulose: Emulsion Component or Standalone Biomaterial?

A direct head-to-head test reviewed here directly compares native BC against acid-hydrolyzed BC nanocrystals as stabilizers for the same olive oil emulsion, addressing whether converting BC into discrete nanocrystals is necessary or beneficial for emulsion applications at all [26].

**Figure 3:**
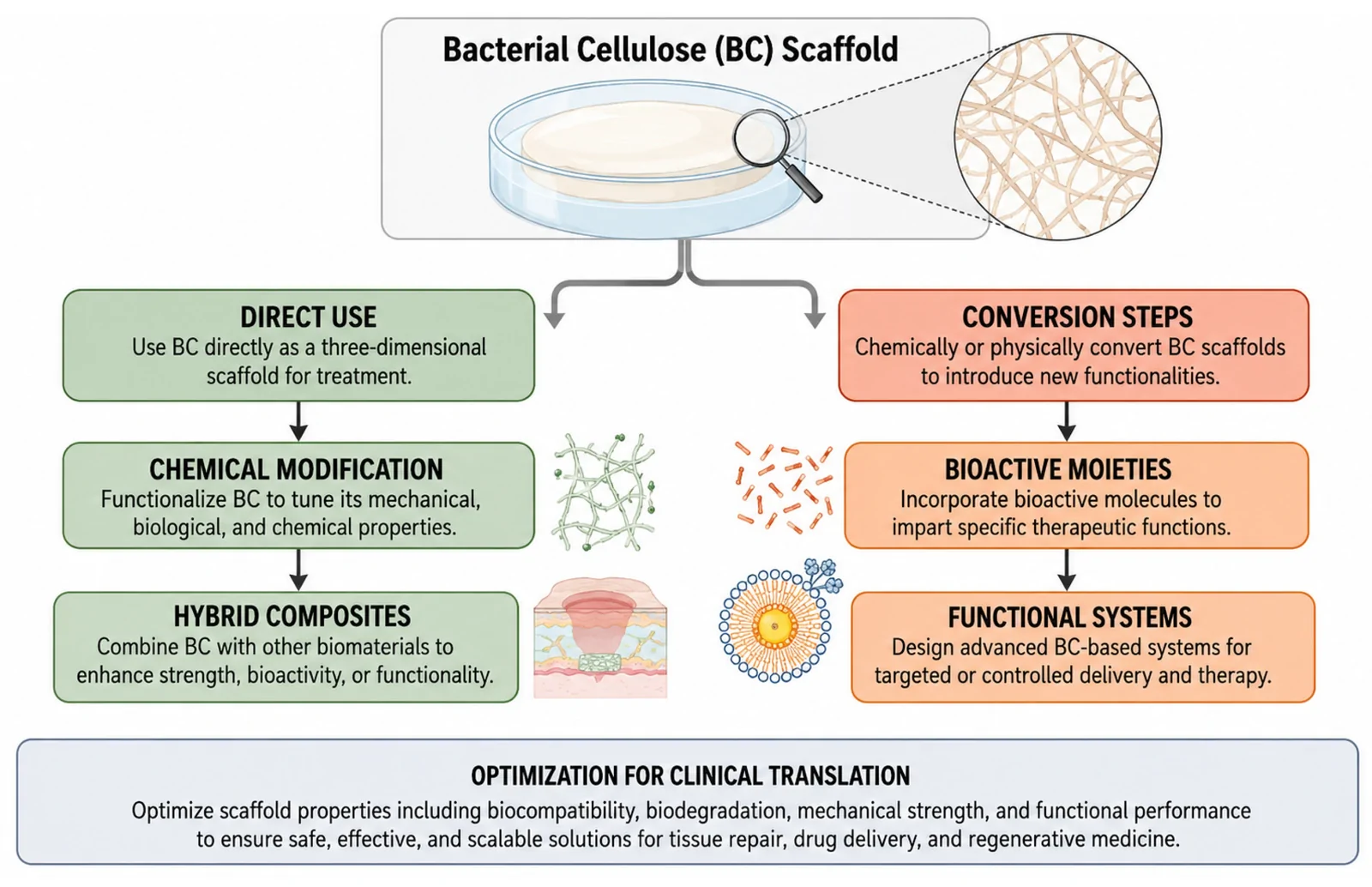
Bacterial cellulose: standalone biomaterial versus Pickering-emulsion stabilizer.

The practical implication is that BC’s route into an emulsion-stabilization role is neither automatic nor free of trade-offs. The conversion step needed to turn a bacterial cellulose pellicle into discrete Pickering particles introduces its own processing variables that can degrade rather than improve emulsifying performance if not carefully controlled, and the resulting BCNC/BCNF is measurably more sensitive to pH and ionic strength than the parent BC network. By contrast, the standalone biomedical applications of BC surveyed in Section 5 and Section 8 exploit exactly the pre-assembled network structure that the emulsion-stabilizer route has to deliberately dismantle. This asymmetry likely explains why BC appears far more often in this review as a standalone material than as the source of the Pickering particle itself, whereas wood-pulp-derived CNCs and CNFs dominate the emulsion-stabilizer role (Table 2). Dedicated studies on bacterial cellulose nanocrystals and nanofibrils as Pickering stabilizers [45,46] document both the performance of this conversion route and its emerging applications. Figure 3 illustrates this branching distinction.

### 2.7. Section Critical Summary

Section 2 establishes a mechanistically coherent picture of how and why cellulose particles stabilize emulsions, but strong evidence is mechanistic rather than translational: almost none of the fundamental-tier studies test a pharmaceutical payload, a physiologically relevant medium, or a manufacturing-relevant scale. Four specific gaps recur: oil-specificity of stabilization is under-characterized (Table 1); supplier-to-supplier CNC variability is documented but rarely controlled for downstream (Table 1); and long-term, physiological-condition stability data are sparse relative to benchtop proof-of-concept studies. Bacterial cellulose specifically requires a non-standardized conversion step before it can serve as a Pickering stabilizer at all, making its emulsion performance less mature than its standalone-biomaterial performance (Section 2.6).

## 3. Emulsions as Templates for Porous Materials and Tissue Engineering

### 3.1. High Internal Phase Emulsions and Porous Scaffolds

When the internal phase volume exceeds ~74%, emulsions become high internal phase emulsions (HIPEs) whose polyhedral droplets, once the continuous phase is solidified, template interconnected macroporous solids [27,30,47]. Nanocellulose is well suited to HIPE stabilization because irreversibly adsorbed particles survive the high shear and osmotic stresses involved [27,32]. Benchmarking and production advances have made CNCs and CNFs available at scales relevant to material manufacture [23,34], and cellulose- and collagen-derived scaffolds have been explored for bone tissue engineering [28,48] (Table 3). A comprehensive critical appraisal of cellulose-templated porous structures, HIPE-derived foams, and tissue engineering scaffolds is summarized in Table 3.

### 3.2. Hydrogels and Composite Carriers from Emulsion Templates

Emulsion and Pickering-emulsion templating also yield hydrogel and composite carriers with tunable—often stimulus-responsive—release, including pH-responsive polyacrylic acid–CMC hydrogels [51], magnetic core–shell CMC hydrogels [54], and cellulose-nanowhisker/starch microhydrogels [50]. As a class, characterization is dominated by physicochemical release testing in buffer, with cell-culture or in vivo confirmation being the exception rather than the rule.

### 3.3. Section Critical Summary

The templating literature convincingly demonstrates that cellulose-stabilized HIPEs can be engineered into macroporous scaffolds with tunable pore architecture, and that surface modification can push structural performance beyond what unmodified particles allow (Table 1). What remains underdeveloped for biomedical—as opposed to materials-engineering—applications is the translational endpoint: cell-adhesion or short-term cytocompatibility data are common, but longer-term in vivo integration, vascularization, degradation kinetics, and immunogenicity data are comparatively scarce.

## 4. Drug Encapsulation and Formulation

### 4.1. Loading and Release Principles

Cellulose emulsions encapsulate lipophilic actives within the oil phase, keeping the drug dissolved and presenting a large interfacial area for absorption [3]. Different cellulose derivatives govern release rate and mechanism, as established in preformulation studies [55,56], and Pickering emulsions have specifically been shown to improve oral drug delivery [3,44,57].

Across these five studies, all are methodologically solid and all report favorable in vitro loading or release profiles, but none reports a matched in vivo pharmacokinetic or pharmacodynamic endpoint for the specific formulation described. This is a general pattern across the cellulose-microsphere literature in this bibliography, and it means the ‘proof’ offered by Table 4 below is, for most rows, proof of controllable in vitro release rather than proof of clinical benefit. One formulation line is a partial exception.

Table 4 provides a detailed critical appraisal of key primary studies investigating cellulose- and nanocellulose-based carriers for drug encapsulation and sustained delivery.

### 4.2. A Partial Exception: A Formulation Line with Documented Follow-Up

Among the drug-encapsulation studies surveyed (Table 4), Chaturvedi and Kulkarni’s [68] PHB/cellulose-acetate-phthalate 5-fluorouracil microspheres stand out for a documented 2013 cytotoxicity and antitumor follow-up study—one of the few cellulose-carrier lines here with a demonstrated progression from physicochemical characterization toward a biological efficacy endpoint. Colon-targeting is nonetheless inferred from in vitro pH-dependent dissolution rather than confirmed in vivo.

### 4.3. Section Critical Summary

Overall, the evidence supports controllable drug release from cellulose-based carriers across a range of formulations, but remains dominated by in vitro characterization; matched in vivo pharmacokinetic and efficacy studies—following the model set by the 5-fluorouracil/PHB line [68]—are the clear next step for the most mature carrier systems.

## 5. Antimicrobial and Pathogen Applications

Antimicrobial applications dominate the cellulose-emulsion literature, comprising the largest single application category among the 142 references retained in this review (48 of 142, approximately one-third of the total). Cellulose- and nanocellulose-stabilized emulsions protect volatile essential oils from evaporation and oxidation, enhance contact with microbial membranes, and enable sustained release from films, fibers, and coatings [70,71,72,73,74].

### 5.1. Essential-Oil Pickering Emulsions: Foundational Studies

Read alongside the wider literature they represent, these studies indicate a pattern: nearly all downstream papers on oregano and cinnamon Pickering emulsions are materials-science extensions—new film matrices, new co-stabilizers, new packaging formats—rather than independent re-tests of antimicrobial potency against clinically relevant pathogens or biofilms.

Romulo et al. [75] found that a 5% thyme-oil emulsion was completely inactive while a 10% formulation crossed into measurable activity is an important corrective to the common secondary-literature claim that Pickering stabilization allows a straightforward, large reduction in effective oil concentration relative to free oil. Whether a given reduction crosses below an efficacy threshold depends on the specific oil, pathogen, and cellulose system, and reported potencies vary by roughly three orders of magnitude across different essential-oil/cellulose systems in this bibliography. Compare Romulo et al. [75] MIC50 in the thousands of μg/mL against the sub-microliter-per-mL potencies reported by Zhou et al. [76], Liu et al. [77], Oun et al. [72], and Thongsrikhem et al. [78].

### 5.2. Delivery Formats, Wound Dressings, and Pathogen Targets

Cellulose emulsions are frequently cast into functional formats: antibacterial films and edible coatings incorporate nanoemulsions or Pickering emulsions of essential oils [79,80,81,82,83,84]; electrospun cellulose-acetate and composite nanofibers embed antimicrobial agents and nanoparticles [70,85,86,87]; and bacterial-cellulose and nanocellulose membranes are functionalized with silver, ZnO, or chitosan [88,89,90,91]. In wound care, bacterial-cellulose dressings support moist wound healing [92], cellulose-acetate/gelatin nanofibrous dressings combine antibacterial action with healing promotion [86], and antibacterial carboxymethyl-cellulose/quaternized-starch and chitosan-cellulose systems have been developed with wound-relevant properties [74,85,93,94]. Table 5 collates wound-oriented studies; Ouyang et al. [95] is examined above because it uniquely benchmarks against an already-marketed clinical product.

Across these studies, the most frequently tested pathogens are the Gram-positive Staphylococcus aureus and the Gram-negative Escherichia coli, with additional work against Pseudomonas aeruginosa, Listeria monocytogenes, Salmonella, Bacillus, and the fungus Candida albicans [70,71,73,76,77]. Topical CNC-stabilized nanoemulgels loaded with ciprofloxacin and controlled-release chlorhexidine systems extend this activity to conventional antimicrobials [116,117].

### 5.3. Section Critical Summary

This is the review’s largest and most internally consistent evidence base, but also its most methodologically homogeneous: the overwhelming majority of studies use essential-oil actives, CNC or CNF stabilization, and food-microbiology-style susceptibility testing against a recurring small panel of laboratory reference strains. Table 5’s six anchor studies are partial exceptions that each break this mold in a different way—a real food-matrix trial [96], a rare antiviral test [97] (though with a rapid-antigen-test readout rather than a gold-standard plaque assay), a concentration-threshold finding [75], and a clinical-product benchmark [95].

## 6. Vaccine and Immunotherapeutic Delivery

Emulsions are established vaccine adjuvants (see Table 6), and particle-stabilized systems are an emerging refinement. This is the smallest section in the review (four curated references), but unusually for this bibliography, it contains one of the most rigorous in vivo efficacy studies available—and a real, unresolved disagreement between two of its four references.

### 6.1. Chlamydia Subunit Vaccine: In Vivo Efficacy

Shu et al. formulated a chitosan-particle-stabilized Pickering emulsion combined with interleukin-12 (IL-12) as an adjuvant/delivery system for a Chlamydia trachomatis Pgp3 subunit vaccine, addressing the limitation that Pgp3 antigen alone elicits insufficient protective immunity (Table 6) [121]. It is one of the few studies in this bibliography with a full in vivo efficacy readout: antibody titers, cytokine profile, and actual pathogen load and pathology reduction after challenge. Two principal limitations apply. First, the evidence is confined to a single antigen, pathogen, and animal species, so generalization to other vaccine targets or to humans remains unestablished. Second, the approximately 790 nm droplet size is considerably larger than the approximately 160 nm optimum identified by the droplet-size benchmark study [118] for immune-cell recruitment, an inconsistency that cannot be resolved from the literature surveyed here and that defines an open research question for this field.

### 6.2. Section Critical Summary

Despite the unresolved inconsistency discussed below, the vaccine application itself is one of the best-evidenced translational examples in this review: Shu et al. [121] achieved robust in vivo protective immunity against genital chlamydial infection using a chitosan Pickering emulsion, one of only a few studies in this bibliography with confirmed animal-model efficacy rather than an in vitro proxy alone. That said, the droplet-size study [118] and Shu et al.’s chitosan Pickering emulsion study [121] contain a real, unresolved inconsistency: the droplet-size study [118] identifies ~160 nm as the superior emulsion droplet size for immune-cell recruitment, while Shu et al. [121] achieved its robust in vivo efficacy with droplets nearly five times larger (~790 nm). Because the two studies differ in particle chemistry, antigen, and species, this discrepancy cannot currently be resolved from the literature reviewed here; it may reflect a genuinely different optimum for particle-stabilized versus surfactant-stabilized adjuvants, or it may indicate that the 160 nm figure does not generalize beyond the specific squalene-emulsion chemistry in which it was established. A dedicated droplet-size optimization study for cellulose- or chitosan-Pickering-emulsion adjuvants specifically—holding antigen and particle chemistry constant while varying only size—does not yet exist and is a clearly identified research priority.

The vaccine evidence base is small but unusually strong for one reference: Shu et al. [121] is one of the few studies in this review reporting antibody titers, cytokine profiles, and post-challenge pathogen reduction in a real animal model. Before any cellulose-nanocrystal-specific adjuvant claim can be made with confidence, a droplet-size optimization study matched to cellulose-Pickering chemistry and replication of the chitosan/IL-12/Pgp3 result with a second antigen remain needed.

## 7. Topical and Transdermal Delivery

Cellulose derivatives have a long history in dermal and pharmaceutical formulation. Comprehensive reviews of cellulose application in pharmaceutical industries [122] and pharmaceutical compounding [123] establish the background: from simple viscosity enhancement to emulsion stabilization, cellulose derivatives have been embedded in topical dosage forms for decades. A broader survey of cellulose sources is provided by Lavanya et al. [124], while the physicochemical properties of cellulose and its derivatives as pharmaceutical excipients are treated in detail by Doelker [125]. The methodological designs, key outcomes, and limitations of primary cellulose-based topical and transdermal delivery systems are systematically compared in Table 7.

### 7.1. Conventional Cellulose in Topical Emulsion Formulation

Before the Pickering era, cellulose derivatives served as secondary stabilizers and viscosity builders in topical creams and multiple emulsions. Microcrystalline cellulose combined with sodium CMC (Avicel CL-611) was specifically developed as a cream-stabilizing system and characterized for its viscoelastic contribution to cream structure [126,131]. The W/O/W multiple-emulsion architecture, examined in a dedicated stability study using cellulose-derivative thickeners in the inner aqueous phase as an approach to dermal delivery of oligonucleotide active pharmaceutical ingredients, established that osmotic balance and cellulose-thickener selection jointly govern multiple-emulsion stability [127]. These pre-Pickering results define the baseline performance that nanocellulose-based Pickering topical systems are now positioned to improve upon. The O/W/O multiple-emulsion format—in which drug transfers through an intermediate oil phase to prolong skin residence relative to a simple emulsion—provides pharmacokinetic rationale for the W/O/W architecture investigated here [131].

#### Multiple-Emulsion W/O/W Architecture for Oligonucleotide Delivery

Schmidts et al. investigated W/O/W multiple emulsions as dermal carriers for oligonucleotide active pharmaceutical ingredients, specifically a DNAzyme targeting inflammatory skin disease [127]. The study screened osmotic-active additives in the inner aqueous phase—NaCl, MgSO4, glucose, and glycine—alongside two cellulose-derivative thickeners, using Span-80 as the primary emulsifier and various hydrophilic co-emulsifiers for the outer aqueous phase. Osmotic balance in the inner phase emerged as the critical stability determinant; cellulose derivatives functioned as secondary viscosity modifiers rather than as primary interfacial stabilizers, and the most promising formulations were confirmed compatible with a DNAzyme oligonucleotide payload. This is a pre-Pickering-era reference (2010) in which cellulose appears as a conventional excipient rather than as a nanoparticle stabilizer. Its value lies as a precedent establishing the clinical utility of W/O/W architecture for dermal delivery of water-soluble macromolecular drugs—a utility that a nanocellulose Pickering multiple emulsion could replicate with superior stability and without synthetic surfactants. Emerging work on CMC-stabilized W/O/W emulsions with pH-responsive behavior and mucoadhesive properties for the co-delivery of active compounds suggests that cellulose-primary multiple-emulsion delivery continues to advance beyond this 2010 baseline, though dedicated cellulose-Pickering multiple-emulsion studies with full pharmacokinetic and mucoadhesion characterization remain to be published.

### 7.2. Nanocellulose-Stabilized Pickering Emulsions for Topical Drug Delivery

Particle-stabilized oil-in-water emulsions provide a versatile topical delivery platform for lipophilic drugs, with one study directly demonstrating their utility for a poorly water-soluble antifungal [129]. Hydrophobically functionalized CNCs have been used as both Pickering emulsion stabilizers and as the basis for nanolatex synthesis—though, as discussed in earlier sections, the nanolatex application is a polymer-chemistry use case rather than a topical delivery system [33]. Ionic-liquid-assisted cellulose emulsions and BC-based formulations round out the portfolio of approaches for permeation enhancement and controlled delivery across or through skin barriers [128,130].

#### CNC-g-PIPP Pickering Emulsion for Topical Drug Delivery

Hiranphinyophat et al. developed a bifonazole-loaded oil-in-water emulsion stabilized by cellulose nanocrystals grafted with a polyphosphoester (CNC-g-PIPP) and evaluated it as a topical antifungal carrier using ex vivo porcine skin [129]. The emulsion had a mean droplet size of 2.54 ± 1.39 μm and remained stable for at least 15 days without significant change. Layer-resolved skin distribution—achieved by trypsin digestion of the stratum corneum followed by separate analysis of viable epidermis and dermis—confirmed improved bifonazole penetration into deeper skin layers, and histopathological examination showed no irritation. This is the strongest topical-delivery primary study in the entire bibliography: an actual nanocellulose Pickering emulsion carrying a poorly soluble drug, tested with layer-resolved permeation and histological irritation assessment. Three limitations define its current stage: the 15-day stability window is short relative to typical pharmaceutical shelf-life requirements, porcine ex vivo skin is a step removed from human in vivo testing, and the CNC surface chemistry requires a synthetic polyphosphoester graft rather than unmodified natural cellulose. A subsequent study from the same group substituting lanoconazole for bifonazole and moving to a live-mouse anti-inflammatory model represents a progressive, iterative advance toward clinically relevant endpoints that is uncommon in the topical-delivery literature surveyed here.

### 7.3. Topical Formulation Overview and Permeation Mechanisms

The stratum corneum remains the principal barrier to topical drug delivery, with transcellular and intercellular lipid pathways as the dominant permeation routes for most drugs. Cellulose-based carriers engage these pathways in two ways: directly, through the oil phase of emulsions solubilizing lipophilic drugs and partitioning into intercellular lipids; and indirectly, through particle-covered droplet surfaces that reduce interfacial tension and may facilitate contact with corneocytes. Ionic liquids combined with cellulose formulations add a third mechanism: temporary disruption of the ordered intercellular lipid lamellae [128]. These multiple mechanisms combine to make cellulose-emulsion topical formulations a genuinely multi-pathway delivery platform, though each mechanism requires individual confirmation for a given drug–skin combination rather than being assumed from the platform literature.

### 7.4. Section Critical Summary

The CNC-g-PIPP Pickering-emulsion line for topical bifonazole and lanoconazole delivery [129] is one of the more rigorously characterized topical results in this review, with clear formulation and stability data supporting its translational promise. The most important corrective finding from this section, however, is the placement of Zhang et al. [33]: it is a polymer-latex synthesis study, not topical-delivery evidence. Once that correction is applied, the primary Pickering-emulsion topical evidence narrows to the CNC-g-PIPP line (Hiranphinyophat et al. [129] and its lanoconazole follow-up), with independent replication with additional drug payloads being the clear next step.

## 8. Commercialized Products and Clinical Translation

Industrial-scale nanocellulose production, bacterial cellulose biomedical platforms, waste-stream valorization, patent landscape, and the colon-targeted microsphere line with documented preclinical follow-up are the five translation themes examined in this section.

### 8.1. Industrial-Scale Production: What Is Actually Available

Industrial-scale nanocellulose production exists but remains concentrated among a small number of producers. CelluForce’s Windsor, Quebec plant is the world’s largest cellulose nanocrystal facility, with a capacity of approximately 300 tonnes per year; other producers include Borregaard (Norway), Melodea (Israel), Blue Goose Biorefineries, and GranBio (Brazil) [23,34]. This scale is sufficient for industrial applications (coatings, cosmetics, oilfield fluids) but modest relative to the volumes a pharmaceutical excipient supply chain would eventually require.

The economic context for the pharmaceutical adoption of nanocellulose as an emulsion stabilizer is not yet fully established. Pharmaceutical-grade CNC is estimated to cost approximately US$10–100 per kilogram at pilot scale, substantially higher than commodity synthetic surfactants (e.g., polysorbate 80 at ~US$3–8/kg) but competitive with specialty pharmaceutical excipients [23,34]. The cost differential is partially offset by the reduced required excipient load in Pickering emulsions (typically 0.1–2 wt% nanocellulose) relative to surfactant-stabilized systems, and by the growing availability of waste-stream cellulose feedstocks—including the cigarette-filter-derived cellulose acetate approach discussed in Section 8.3—which could substantially reduce raw-material costs if extraction/purification protocols are validated for pharmaceutical quality. The economic viability of nanocellulose-based emulsion products will ultimately depend on demonstrating a cost-per-quality-adjusted outcome advantage over existing formulations, a calculation that requires the in vivo efficacy and comparative-effectiveness data whose absence is a recurring gap throughout this review.

### 8.2. Bacterial Cellulose: The Most Mature Biomedical Cellulose Platform

Bacterial cellulose occupies a unique position in the commercialization landscape: it is simultaneously the most biomedically advanced cellulose material (with established clinical use as wound dressings and documented applications in regenerative medicine) and the most expensive and limited in throughput, due to its fermentation-based production route.

Gorgieva’s comprehensive review of bacterial cellulose modification routes (in situ during biosynthesis, and ex situ post-synthesis treatment) as a platform for multifunctional biomedical materials, covering the 2010–2020 literature, confirms BC’s established properties—high crystallinity, nanofiber-scale diameters, exceptional water-holding capacity, and non-cytotoxicity from the absence of lignin and hemicellulose—across applications spanning wound healing, artificial skin, vascular grafts, nerve coverings, and cartilage. It identifies two limitations directly relevant to this section: structural compactness restricts cell infiltration without modification, and slow in vivo degradation is advantageous for durable implants but problematic for resorbable wound dressings. As in Section 2.6, this review does not address BC in Pickering-emulsion contexts, reinforcing the standalone-material-versus-stabilizer distinction maintained throughout this bibliography. Together with the 2017 Moniri review [132], it shows that by 2020 BC modification routes had matured substantially while commercial uptake in high-value medical applications remained at the proof-of-concept stage—consistent with this review’s overall finding that translational gaps, not material performance, are the binding constraint.

### 8.3. Waste-Stream Valorization: Cellulose Acetate from Cigarette Filters

A less conventional but commercially significant translation pathway is waste-stream valorization, where the raw material for biomedical cellulose products is extracted from a high-volume waste source rather than virgin biomass.

Khan et al. recovered cellulose acetate from cigarette-filter waste via acid and solvent extraction, electrospun it into nanofibrous membranes, and demonstrated controlled release of tetracycline hydrochloride as a proof-of-concept for biomedical utility, with FTIR, SEM, TGA, and XRD confirming successful recovery of functional CA [133]. The sustainability rationale holds up: cigarette filters are produced at approximately 750 billion units per year globally, and filter-grade CA is a standardized polymer that could serve as a low-cost feedstock for pharmaceutical-grade nanofibers. Two gaps limit immediate translation: batch-to-batch composition variability across filter manufacturers is not addressed in this initial study, and full characterization of extractable/leachable contaminants (residual plasticizers, tobacco-derived compounds) from the original filter matrix—a regulatory prerequisite—has not yet been reported.

### 8.4. Patent Landscape and Intellectual Property Status

The patent landscape for cellulose nanocrystals as of 2012 [134], discussed in Section 2, captured only the early foundational IP wave. The period 2018–2025, not covered by that survey, has seen substantial additional filing activity—specifically around Pickering-emulsion formulations, antimicrobial coatings, and vaccine-adjuvant applications. A dedicated updated freedom-to-operate analysis is warranted for any group pursuing commercial development of cellulose-emulsion biomedical products, as the post-2018 patent landscape is substantially more complex than the 2012 baseline would suggest.

### 8.5. Cellulose Acetate Phthalate and Colon-Targeted Drug Delivery: A Line with Documented Follow-Up

Chaturvedi and Kulkarni’s [68] PHB/cellulose-acetate-phthalate 5-fluorouracil microsphere line, discussed in Section 4.2, is one of the few cellulose-carrier formulations reviewed here with a documented preclinical cytotoxicity/antitumor follow-up, making it a strong near-term oral commercial translation candidate. Table 8 outlines a critical appraisal of manufacturing, waste valorization, and commercialization-relevant studies across the cellulose translation pipeline.

### 8.6. Section Critical Summary

The commercialization section’s evidence base contains a notable asymmetry: a clear and most detailed commercial progress is documented for bacterial cellulose in wound care and regenerative medicine [130,132,135], but this is BC as a standalone non-emulsion material, not as a Pickering-emulsion stabilizer. The two specific lines with strong near-term pharmaceutical translation rationales are: (i) the PHB/cellulose-acetate-phthalate colon-targeted microsphere line [68], which has a documented preclinical follow-up, and (ii) the CNC-g-PIPP Pickering topical emulsion line [129].

## 9. Regulatory Gaps and Shortcomings

Despite mature underlying science, several gaps impede clinical and commercial adoption. The characterization and reproducibility of nanocellulose remain non-standardized, complicating regulatory review. Safety and performance data for specific dosage forms exist but are fragmented across formulations and endpoints, as discussed in Section 4.1 [66,67], where strong in vitro characterization was not matched by in vivo or clinical data—precisely the pattern regulators would need addressed before considering a cellulose-emulsion-based product for approval.

### 9.1. Regulatory Standards, Pathways, and Existing Frameworks

Regulatory infrastructure for nanocellulose is emerging but incomplete. Standardization efforts are underway through ISO Technical Committees 229 (Nanotechnologies) and 6 (Paper, board and pulps), which have published terminology and characterization standards including ISO/TS 20477:2017 (standard terms and definitions for cellulose nanomaterials), ISO/TR 19716:2016 (characterization of cellulose nanocrystals), and ISO/TS 16195:2018 (test-material specifications), alongside the Canadian Standards Association’s CSA Z5100-14 test methods and TAPPI’s International Nanocellulose Standards Coordination Committee nomenclature efforts. These address terminology and basic characterization but do not yet constitute a pharmacopeial monograph or a dedicated FDA or EMA guidance document specific to nanocellulose as a drug product component. By contrast, conventional (non-nano) cellulose derivatives—such as microcrystalline cellulose, hydroxypropyl methylcellulose, ethylcellulose, and carboxymethylcellulose sodium—are long-established, compendial excipients listed in the FDA Inactive Ingredient Database and recognized in USP/NF, Ph. Eur., and other pharmacopeias for oral use, though microcrystalline cellulose specifically is not approved for parenteral administration. Nanocellulose forms (CNC, CNF, and BC-derived nanomaterials) have no equivalent monograph or Inactive Ingredient Database listing, meaning a cellulose-emulsion product using these materials as functional components would likely require a full nanomaterial characterization package under the FDA’s nanotechnology guidance or the EMA’s reflection paper on nanotechnology-based medicinal products, rather than being able to rely on the established safety history of conventional cellulose excipients. Closing this pharmacopeial gap, rather than developing new cellulose chemistries, is likely the single highest-leverage regulatory step for the field.

Darbasizadeh et al. [137] PVA-CMC/ZnO/erythromycin nanofiber illustrates the safety-characterization gap directly: it demonstrates antibacterial efficacy but, based on the available material, reports no quantitative residual-crosslinker analysis (e.g., HPLC or GC-MS quantification of unreacted glutaraldehyde) and no cell-based cytotoxicity assay (e.g., MTT or CCK-8 on human dermal fibroblasts). This is exactly the kind of multifunctional composite system that needs dedicated safety evaluation before any biomedical claim can be considered complete. The core regulatory, characterization, and safety gaps currently hindering the clinical adoption of cellulose emulsion products are highlighted in Table 9.

From a clinical and patient-outcome perspective, the case for cellulose Pickering emulsions ultimately rests on unmet medical needs that existing formulations do not adequately address. For poorly soluble oral drugs, improved bioavailability means lower doses, reduced gastrointestinal side effects, and more predictable pharmacokinetics—directly benefiting patients with chronic conditions requiring long-term therapy. For wound-care antimicrobials, extended-release Pickering dressings could reduce dressing-change frequency in chronic wounds, improving patient comfort and reducing healthcare-associated infection risk. For vaccine adjuvants, particle-stabilized emulsions offer the prospect of single-dose protective immunity for neglected tropical diseases where cold-chain requirements limit conventional adjuvant access. Each of these patient-outcome arguments requires clinical-trial-level evidence to substantiate, reinforcing the review’s consistent finding that translational evidence, not materials-science performance, is the binding constraint.

### 9.2. Section Critical Summary

The regulatory gaps identified here are not primarily about whether cellulose emulsions work—Section 4, Section 5, Section 6 and Section 7 establish, with reasonable, and in places strong, confidence, that they can encapsulate drugs, deliver antimicrobial payloads, and in several documented cases achieve real in vivo, realistic-matrix, or preclinical-follow-up efficacy. The gap is about the completeness and standardization of the evidence package. Batch-to-batch particle characterization, in vivo confirmation of in vitro release performance, biofilm- and resistance-relevant antimicrobial testing, resolution of the droplet-size discrepancy in adjuvant systems, independent replication of strong topical-delivery results, multifunctional-composite safety data, and an updated patent landscape are the specific, addressable items standing between the current literature and a viable regulatory submission. To summarize the analytical scope of this review, Table 10 presents a quantitative breakdown of literature coverage and primary study appraisal depth across all eight thematic domains.

## 10. Conclusions and Research Priorities

**Figure 4:**
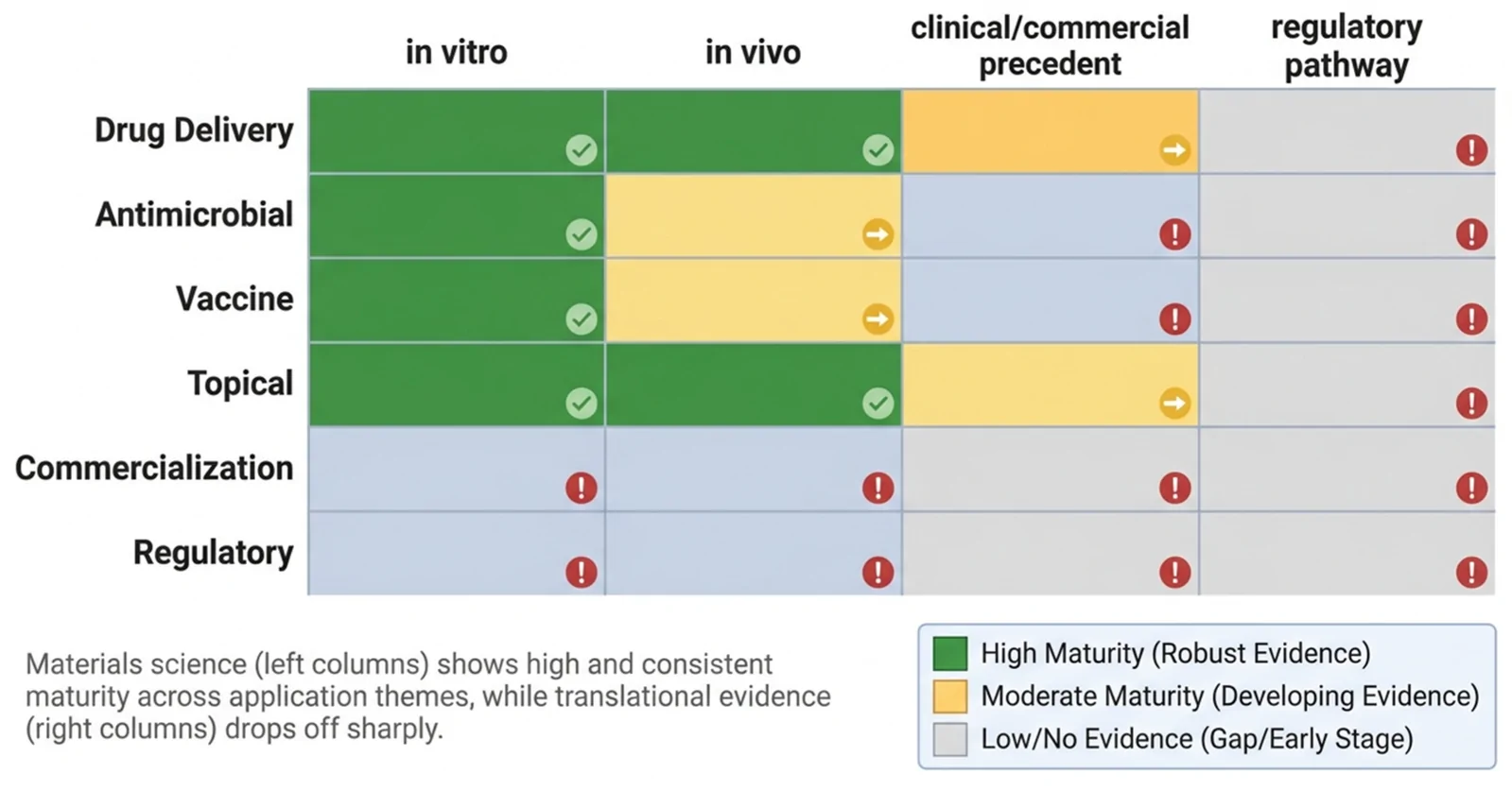
Evidence-maturity matrix across application themes, from in vitro to regulatory approval. Each application theme (radial axis) is scored on a five-level evidence-maturity scale: 1 = in vitro proof of concept only; 2 = in vitro plus physicochemical characterization; 3 = ex vivo or preclinical (animal) data available; 4 = early-phase human or clinical pilot data; 5 = regulatory submission or approved product. Scores represent the highest level of evidence documented across the primary studies reviewed for each theme, as assessed by the structured critical-appraisal methodology described in the Section Literature Search Strategy. No theme currently reaches level 5; the dashed outer ring indicates the target for clinical translation.

This review has synthesized evidence across eight application themes, critically appraising 37 primary studies in depth through structured tables and examining the remaining literature at the pattern level. Cellulose and nanocellulose Pickering emulsions offer superior stability, biocompatibility, and sustainability relative to synthetic-surfactant systems. Demonstrated applications include drug encapsulation and controlled release; antimicrobial formulations validated in both benchtop and real food-matrix conditions [76,96]; an unusually well-evidenced vaccine-adjuvant application [121]; a rigorously characterized topical antifungal carrier [129]; a colon-targeted oncology formulation line with documented preclinical follow-up [68]; a hemostatic wound material benchmarked against a marketed clinical product [95]; and structural templates for tissue engineering and materials engineering [25,27,28]. A key distinction, developed in Section 2.6, is that bacterial cellulose functions primarily as a standalone biomedical material rather than as a Pickering-emulsion stabilizer in the sense used throughout this review. Converting it to nanocrystals or nanofibrils for Pickering use involves a deliberate disintegration step that is not yet standardized and trades some environmental robustness for processability. The review’s overall assessment is that the fundamental materials science of cellulose Pickering emulsions is mature and consistent, while the translational evidence—in vivo confirmation, biofilm-relevant antimicrobial testing, standardized characterization, resolved mechanistic inconsistencies, multifunctional-composite safety data, and up-to-date intellectual property mapping—remains as the binding constraint on clinical and commercial adoption. Figure 4 visualizes this evidence-maturity pattern across the six application themes.

### 10.1. Research Priorities

Six priorities emerge from this evidence, each tied to a specific gap identified in the sections above. First, standardized nanocellulose characterization, release-testing, and long-term/shelf-life stability protocols are needed to control for supplier-to-supplier CNC variability and enable direct comparison across cellulose sources (Section 2). Second, the field’s strong in vitro drug-release science must be paired with matched in vivo pharmacokinetic and efficacy studies; the 5-fluorouracil/PHB microsphere line [68] demonstrates how such follow-up can be structured (Section 4). Third, antimicrobial evaluation must shift from food-microbiology-style planktonic assays [142] toward biofilm- and resistance-relevant testing against clinically sourced pathogens, building on the real-matrix precedent set by Xu et al. [96] and the clinical-product benchmarking by Ouyang et al. [95] (Section 5). Fourth, the droplet-size discrepancy between the squalene emulsion adjuvant benchmark and the chitosan Pickering emulsion vaccine result must be resolved with a dedicated, particle-chemistry-matched size-optimization study (Section 6). Fifth, the CNC-g-PIPP Pickering emulsion topical platform [129] should be independently replicated with additional drug payloads and advanced toward human skin studies (Section 7). Sixth, dedicated safety data for multifunctional composite carriers—including crosslinker residues and metal-oxide/antibiotic combinations—and an updated patent landscape are needed before any of these systems can be submitted for regulatory review (Section 8 and Section 9). Taken as a whole, the evidence indicates that cellulose-based emulsions have progressed beyond proof-of-concept materials and now constitute a credible translational platform for advanced biomedical delivery systems. Future progress will depend less on discovering new cellulose chemistries than on generating standardized, reproducible, and clinically relevant evidence capable of supporting regulatory approval.

Three further directions merit specific attention as the field matures. First, personalized medicine: stimuli-responsive Pickering emulsions that release drug payloads in response to patient-specific microenvironmental cues—tumor pH, inflammatory ROS, or skin-barrier disruption markers—represent a natural next step for the pH- and temperature-responsive cellulose-nanofiber systems documented in Section 4, but require individual patient characterization data to define trigger thresholds. Second, inhalation delivery: oil-in-water cellulose Pickering nanoemulsions with droplet diameters of 100–500 nm are mechanistically suited to nebulization for the pulmonary delivery of lipophilic bronchodilators or antimicrobials against ventilator-associated pneumonia, extending the antimicrobial platform discussed in Section 5 to the respiratory tract; the key design challenge is demonstrating pulmonary surfactant compatibility and avoiding alveolar inflammation. Third, integration with 3D printing: cellulose Pickering emulsions have been proposed as bioinks for extrusion-based bioprinting, where the yield-stress rheology and shear-thinning behavior that confer Pickering stability also provide the printability window needed for filament formation; coupling this with the HIPE-templating scaffold work reviewed in Section 3 could yield directly printable, drug-eluting wound-care scaffolds as a medium-term commercial target.

### 10.2. Broader Technological and Regulatory Directions

Beyond the six evidence-specific priorities above, several broader technological and regulatory shifts could accelerate translation. AI- and machine-learning-guided formulation optimization, already used elsewhere in pharmaceutical development to navigate large excipient/process design spaces, could substantially compress the empirical screening that currently dominates cellulose-emulsion formulation studies (Table 1). Continuous manufacturing and GMP-scale nanocellulose production, building on existing industrial capacity such as CelluForce’s commercial CNC plant (Section 8.1), would need to be paired with the batch-to-batch characterization standards discussed in Section 9.1 to support regulatory filings. On the regulatory side, the ISO and TAPPI standardization efforts already underway (Section 9.1) provide a foundation that could be extended toward a dedicated pharmacopeial monograph for nanocellulose, analogous to the existing microcrystalline cellulose monograph, which would meaningfully lower the regulatory barrier identified throughout this review. Finally, the multifunctional composite systems discussed in Section 9 (antimicrobial-plus-structural, drug-plus-imaging) point toward a longer-term trajectory of precision-nanomedicine applications (stimuli-responsive or targeted Pickering emulsions) that remain largely unexplored in the cellulose-specific literature reviewed here and represent a natural extension once the foundational evidence gaps identified above are addressed.

## Figures and Tables

**Table 1:** Critical appraisal of fundamentals, manufacturing, and templating studies.

Topic	System Type	Goal	Design	Key Result	Limitation/Gap	Ref.
Foundations	Pickering emulsion (particle-general; review)	Effect of particle properties on Pickering-emulsion preparation and behavior	Narrative review	Particle location, size, wettability and surface chemistry govern droplet stability; particle-stabilized emulsions show lower toxicity and stimuli-responsiveness vs. surfactant systems	Review only; no comparative quantitative stability data across particle types	[8]
Foundations	Pickering emulsion (biological particles; review)	Foams/emulsions stabilized by biologically derived particles (cellulose, lignin, chitin, starch, protein)	Narrative/perspective review	Contact angle (relative to 90°) determines O/W vs. W/O partitioning across all biological particle types	Mechanistic principles drawn mainly from inorganic-particle studies; limited direct confirmation for CNCs	[21]
Foundations	CNC-poly(NIPAM) Pickering O/W	Thermo-responsive (poly(NIPAM)-grafted CNCs) Pickering emulsion, heptane/water	Primary experimental study	0.05–0.5 wt% CNC-poly(NIPAM): O/W stable > 6 months; breaks on heating above LCST; bare CNCs failed to stabilize the same system	Single model oil; no drug payload or cytotoxicity data; synthetic graft undercuts ‘fully biobased’ claim	[17]
Foundations	CNF + CNC Pickering O/W (depletion-stabilized)	CNF (non-adsorbing) + CNC (adsorbing) combined depletion-stabilized Pickering emulsion	Primary experimental study	3 concentration regimes: creaming (low CNF), flocculation (mid CNF), stable micron droplets (high CNF, from 0.1% CNF)	Model/food oils only; no drug payload; no multi-month or physiological-temperature data	[22]
Manufacturing	Cellulose nanocrystals (industrial-scale; review)	Benchmarking review: CNCs transition from lab-scale to industrial production	Benchmarking/critical review	Multiple producers (e.g., CelluForce, 1-tonne/day) by mid-2010s; CNC properties highly sensitive to surface charge/size, which vary by supplier	Supplier-to-supplier CNC variability rarely controlled for in downstream biomedical studies	[23]
Manufacturing	Cellulose nanofibrils (enzymatic pretreatment; non-emulsion)	LPMO enzymatic pretreatment as a greener route to cellulose nanofibrils (CNF)	Primary experimental study	LPMO releases C1-oxidized oligosaccharides without altering fiber morphology; subsequent shearing yields gel-like nanoscale cellulose	Materials/process-chemistry only; not tested in emulsion, drug delivery, or antimicrobial context	[24]
Materials engineering	CNF Pickering O/W (aerogel-templated)	CNF/emulsion composite aerogel with quasi-closed pores (thermal insulation)	Primary experimental materials study	Thermal conductivity as low as 15.5 mW/(m·K); density down to 11.4 mg/cm^3^; high specific modulus and flexibility	Materials/energy application, not biomedical; no biocompatibility or degradation data	[25]
Bacterial cellulose	Bacterial cellulose/BC-nanocrystal Pickering O/W	Native BC vs. acid-hydrolyzed BC nanocrystals (BCN) as olive-oil Pickering stabilizers	Primary experimental study	Both BC and BCN adsorb at interface and resist coalescence; BCN more sensitive to pH/ionic strength than native BC	Conversion to BCN trades environmental robustness for processability; no standardized protocol	[26]
Scaffolds	CNC-UPy Pickering HIPE	UPy-grafted ‘supramolecular’ CNC as HIPE stabilizer for macroporous hydrogel	Primary experimental materials study	CNC-UPy HIPE: 80% internal phase, long-term stability, outperforms unmodified CNCs; monoliths support cell adhesion	Synthetic UPy graft (not fully bio-based); no in vivo integration or degradation data	[27]
Scaffolds	Bacterial-cellulose composite scaffold (review)	BC-based composite scaffolds and BCNC/BCNF as reinforcing agents (review)	Narrative review	BC purity/biocompatibility support scaffold platforms for wound healing, tissue engineering, drug delivery, cancer	No unified comparative framework for mechanical performance; mostly short-term/in vitro data across cited studies	[28]

**Table 2:** Fundamentals reference: emulsion types and cellulose/nanocellulose forms.

Type/Form	Stabilizer/Production Route	Application/Key Attribute	Refs.
Oil-in-water (O/W)	CNC, CNF, MC, HPMC, CMC	Oral delivery, creams, lotions	[6,29,31]
Water-in-oil (W/O)	Hydrophobized microfibrillated cellulose	Lipophilic drug protection, transdermal	[15]
High internal phase (HIPE)	Supramolecular CNC, CNF	Scaffolds, aerogels, foams	[27,30,32]
Nanoemulsion/nanolatex	Hydrophobically functionalized CNC	Polymer latex synthesis (see Section 7 note)	[33]
Pickering (particle-stabilized)	CNC, CNF, BC, modified forms	All applications; review focus	[7,8,16]
Redispersible/dried Pickering	Nanocrystalline cellulose	Powdered emulsion products	[29,31]
Cellulose nanocrystals (CNCs)	Acid hydrolysis of pulp/biomass	Rod-like, crystalline, tunable surface charge	[8,29,34]
Cellulose nanofibers (CNFs)	Mechanical/enzymatic fibrillation	High aspect ratio, entangled networks	[22,24,35]
Bacterial cellulose (BC)	Fermentation (Komagataeibacter)	Ultra-pure, biomimetic network	[26,36,37]
Amphiphilic/modified cellulose	Surface grafting, esterification	Tunable hydrophobicity, responsiveness	[38,39,40]
Regenerated/amorphous cellulose	Dissolution–regeneration	Alternative interfacial behavior	[4,41]
Agricultural-residue nanocellulose	Acid/mechanical from waste	Low-cost, sustainable feedstock	[42]

**Table 3:** Cellulose-templated porous materials and scaffolds for biomedical use.

Material/Structure	Cellulose Component	Target Application	Refs.
HIPE-templated foams	Supramolecular CNC	Porous scaffolds, carriers	[27,30]
Nanocellulose aerogels	CNF, plant nanocellulose	Insulation (Section 2.5), tissue scaffolds	[25,49]
Bone tissue scaffolds	Cellulose/collagen, BC composite	Bone regeneration	[28,48]
Hydrogel–microhydrogel composites	CNC/starch, CMC	Drug-loaded scaffolds	[50,51]
Scaffold-embedded microparticles	Ethyl cellulose microparticles	Drug-eluting scaffolds	[52]
Contact-lens/implant hydrogels	Cellulose-based hydrogels	Ocular implantation	[53]

**Table 4:** Drugs and bioactives formulated in cellulose-based emulsions; detailed appraisal where primary studies were reviewed.

Drug/Bioactive	System Type	Study Focus	Design	Key Result	Gap/Limitation	Ref.
Curcumin	CNC/Tween-20 nanoparticles; Pickering	Curcumin loading onto CNC via surfactant (Tween 20) vs. methanolic medium	Primary experimental in vitro study	Tween/CNC: loading 7.73 mg/g vs. 3.35 mg/g (methanolic); SGF release 8.99 vs. 2.65 mg/L; curcumin activity retained post-release	In vitro only; surfactant-dependent (not surfactant-free); no oral bioavailability data	[58,59]
Ibuprofen	CNF/ChNF Pickering O/W (pH-responsive)	pH-responsive, surfactant-free ibuprofen release via CNF/ChNF Pickering emulsion	Primary experimental study	CNF + ChNF interfacial layer gives long-term emulsion stability with tunable pH-triggered release	In vitro release kinetics only; no in vivo bioavailability or efficacy data	[60]
Glipizide	Ethyl cellulose microspheres	12 h sustained-release glipizide microspheres (ethyl cellulose:Eudragit S100)	Primary experimental study	Optimal batch F3 (2:3 EC:Eudragit S100): ~12 h release; Korsmeyer–Peppas/zero-order fit (*n* = 0.960, anomalous diffusion)	In vitro dissolution only; no in vivo pharmacokinetic or hypoglycemic data for this formulation	[61,62]
Salicylic acid	Ethyl cellulose microspheres	Sustained release	Primary experimental study; quasi-emulsion solvent diffusion (QESD): ethyl cellulose + salicylic acid in dichloromethane (internal phase), PVA/water (external phase); PEG-modified variant included	Microspheres 5–40 μm; PEG addition (ECPSA) eliminated burst release, giving sustained release over 48 h reaching saturation by day 5; release fit Higuchi or Korsmeyer–Peppas models depending on formulation; no cytotoxicity difference vs. control	In vitro release and cytotoxicity only; no in vivo data	[63]
Salbutamol sulphate	Ethyl cellulose microcapsules	Encapsulation efficiency	Primary experimental study; emulsion-solvent evaporation with Tween 80 emulsifier and light liquid paraffin oil phase; microcapsules also compressed into tablets	Microcapsule size decreased with increasing drug loading; biphasic release-immediate from microcapsules, burst then 8 h sustained release from compressed tablets; zero-order and Higuchi kinetics best fit	In vitro dissolution only; no in vivo pharmacokinetic data	[64]
Glibenclamide	Cellulose acetate microparticles	Emulsion-solvent preparation	Primary experimental study; cellulose acetate microparticles via emulsion solvent evaporation; characterized by particle size, encapsulation efficiency, FTIR, DSC, and SEM	Spherical, free-flowing microparticles with stable drug–polymer character (no adverse interaction) and sustained in vitro glibenclamide release	In vitro characterization only; no in vivo or comparative bioavailability data	[65]
Nifedipine/verapamil	Ethyl cellulose/cellulose acetate microspheres (solvent evaporation)	Nifedipine/verapamil microspheres—ethyl cellulose (EC) vs. cellulose acetate (CA) matrix	Primary experimental study	EC gave smaller, narrower particle-size distribution than CA; DSC confirmed molecular drug dispersion; successful tableting	Particle/tableting characterization only; no dissolution-linked bioavailability or in vivo blood-pressure data	[66]
Prednisolone (± HP-β-CD complex)	Cellulose matrix microspheres (oil-in-oil emulsion)	24 h sustained-release prednisolone microspheres (±HP-β-CD complex, cellulose matrix)	Primary experimental study	Optimized batch MIC5 (PRD–HP-β-CD complex): 95.81% release over 24 h	In vitro release only; no anti-inflammatory efficacy or systemic prednisolone exposure data	[67]
5-Fluorouracil	PHB/cellulose acetate phthalate microspheres	pH-responsive release; documented cytotoxicity follow-up	Primary experimental study; solvent-evaporation PHB:CAP blend microspheres (2:1, 1:1, 1:3 *w*/*w*); 29–67 μm; FTIR/DSC characterization; documented 2013 cytotoxicity/antitumor follow-up study	Spherical microspheres with molecular-level 5-FU dispersion and no adverse drug–polymer interaction; pH-sensitive, colon-targeted release profile; follow-up study confirmed cytotoxicity and antitumor activity	Colon-targeting inferred from in vitro pH-dependent dissolution, not confirmed in vivo; follow-up remains preclinical, not clinical	[68]
Ocular actives	Cellulosic polymers	Bioavailability enhancement	Narrative review	Surveys cellulosic polymers (HPMC, CMC, ethylcellulose, methylcellulose, hydroxyethyl cellulose) for solubility enhancement and sustained release across ocular delivery barriers	Review only; no primary experimental or quantitative comparative data	[69]

**Table 5:** Cellulose-emulsion antimicrobial and wound-care systems; detailed appraisal where primary studies were reviewed.

System Type	Goal	Key Result	Limitation/Gap	Ref.
CNC Pickering O/W	Oregano essential oil (OEO) Pickering emulsion stabilized by CNCs	Stability improves with higher CNC/pH, lower oil ratio/salt; antimicrobial efficacy retained—foundational for 6–7 downstream studies	Basic zone-of-inhibition/turbidity assays only; ionic-strength/pH sensitivity limits physiological translation	[76]
CNC Pickering O/W + chitosan film	Cinnamon oil (CEO) CNC-Pickering emulsion embedded in chitosan film	CNC restores mechanical strength lost from added oil; controllable/slow CEO release; good activity vs. food-relevant strains	Food-spoilage pathogen panel, not clinical wound pathogens; no biofilm data	[77]
CNC Pickering O/W	CNC-stabilized thyme oil (TEO) Pickering emulsion at 5% vs. 10% loading	10% TEO: MIC50 = 4096 μg/mL (*S. aureus*, *E. coli*); 5% TEO: no measurable activity—concentration threshold, not smooth dose–response	MIC50 far higher than other oregano/cinnamon systems in this bibliography; potency not generalizable across oils	[75]
Bacterial-cellulose Pickering O/W	Ultrasound-modified BC/thyme-oil (TEO) emulsion coating for chilled chicken shelf life	0.5% BC/TEO-E: lowest bacterial/coliform counts over 12 days; outperforms plain-BC and Tween-surfactant controls	Food-spoilage/refrigeration endpoint, not clinical wound-pathogen or biofilm model	[96]
CNF Pickering O/W	CNF-stabilized tea tree oil (MaEO) Pickering emulsion—eco-friendly disinfectant	Antibacterial vs. *S. aureus* and others; inactivates SARS-CoV-2 (rapid antigen-test readout)	Antiviral endpoint is a rapid-antigen surrogate, not plaque/TCID50; disinfectant framing, not therapeutic wound use	[97]
CNC/alginate microspheres (inverse emulsion)	CNC/alginate/ε-polylysine porous microspheres—hemostatic + antibacterial wound material	High porosity/absorption; hemostasis and antibacterial performance favorable vs. commercial chitosan powder (in vivo)	CNC acts as structural filler here, not as an emulsion-droplet stabilizer in the sense used elsewhere in this review	[95]
Cinnamon, clove, lemon, ginger, eucalyptus, tea tree, black cumin, Satureja, Mentha—CNC/CNF Pickering, composite films	Antibacterial/antifungal packaging, coatings, and active films	Broad materials-science diversification: essential oils (cinnamon, clove, lemon, ginger, eucalyptus, tea tree, black cumin, Satureja, Mentha) incorporated into CNC/CNF Pickering emulsions and composite films, demonstrating antibacterial/antifungal activity across formats	Nearly all downstream studies are materials-science extensions (new film matrices, co-stabilizers, packaging formats) rather than independent re-tests of antimicrobial potency against clinically relevant pathogens or biofilms; testing concentrated on a small panel of standard laboratory reference strains	[73,74,79,80,85,98,99,100,101,102,103,104,105,106,107,108,109,110,111,112,113]
BC dressings, cellulose-acetate/gelatin nanofibers, CMC composites, silver-cellulose systems	Moist wound healing, hemostasis, antibacterial wound support	Bacterial-cellulose dressings support moist wound healing; cellulose-acetate/gelatin nanofibrous dressings combine antibacterial action with healing promotion; antibacterial CMC/quaternized-starch and chitosan-cellulose systems developed with wound-relevant properties	Predominantly in vitro/benchtop characterization; limited head-to-head benchmarking against marketed clinical wound-care products (Ouyang et al. [95] is the notable exception, examined separately)	[86,92,93,94,114,115]

**Table 6:** Critical appraisal of vaccine-adjuvant studies.

System Type	Goal	Design	Key Result	Limitation/Gap	Ref.
Conventional surfactant emulsion (squalene, non-cellulose)	Squalene emulsion-adjuvant droplet size: 20 nm vs. 160 nm (influenza)	Primary experimental in vivo study	160 nm outperforms 20 nm: greater immune-cell recruitment, antigen uptake, faster lymph-node translocation, better response	Conventional surfactant emulsion (MF59-type), not cellulose/Pickering; benchmark value, not direct evidence for CNC systems	[118]
Chitosan particles-in-oil emulsion (non-cellulose)	Solvent-free ‘green’ particles-in-emulsion (quaternized chitosan) adjuvant, 2 veterinary antigens	Primary experimental study	No organic solvents/crosslinkers needed; designed to engage both antibody- and cell-mediated immunity	Conventional oil + chitosan, not cellulose; veterinary antigens (PRRS, FMD-VLP), not human vaccine data	[119]
Multiple carrier types (review)	Classification review of vaccine-adjuvant delivery carriers (VADS)	Narrative/classification review	Positions polysaccharide-Pickering emulsions as one class among lipid NPs, virosomes, OMVs, alpha-glucan particles	Broad-scope review; cellulose/chitosan systems receive brief treatment relative to more advanced platforms	[120]
Chitosan Pickering emulsion + IL-12 (non-cellulose)	Pgp3 subunit vaccine adjuvant for Chlamydia trachomatis; full in vivo efficacy readout	Primary experimental in vivo study; mouse genital chlamydial infection/challenge model; 789 ± 44 nm droplets, +33.2 ± 3 mV surface, ~90% antigen adsorption	Elevated Pgp3-specific IgG/IgG1/IgG2a/IgA and IFN-γ/IL-4; reduced chlamydial load and pathology vs. controls	Chitosan, not cellulose; single antigen/pathogen/species; ~790 nm droplet size inconsistent with the ~160 nm optimum from [118]	[121]

**Table 7:** Critical appraisal of topical and transdermal cellulose-emulsion studies.

System Type	Goal	Design	Key Result	Limitation/Gap	Ref.
MCC/CMC topical cream (Avicel CL-611)	Viscoelastic characterization of topical creams stabilized by MCC/CMC	Primary experimental study	MCC/CMC increases continuous-phase viscosity without excess oil; Avicel CL-611 confirmed as effective cream stabilizer	Pre-nanocellulose; no skin permeation or in vivo efficacy data	[126]
W/O/W multiple emulsion + cellulose-derivative thickener (conventional surfactant)	Dermal W/O/W carrier for oligonucleotide API: osmotic-agent and cellulose-thickener effects on stability	Primary experimental study	Osmotic balance critical; cellulose thickeners improve stability; promising formulations compatible with DNAzyme API	2010 pre-Pickering; cellulose as secondary thickener, not primary stabilizer; advanced by 2025 CMC-primary W/O/W study	[127]
Ionic-liquid/cellulose emulsion	IL-assisted topical delivery: permeation enhancement strategies and toxicology	Narrative review	IL/BC composites improve transdermal flux; safety is concentration- and cation-type-dependent	Limited primary IL/cellulose-Pickering data; IL toxicity at elevated concentrations a concern	[128]
CNC-g-PIPP Pickering O/W	Bifonazole topical/transdermal delivery from CNC-Pickering emulsion; skin layer-resolved permeation	Primary experimental study (ex vivo porcine skin)	2.54 ± 1.39 μm droplets; stable ≥ 15 days; improved deep-skin penetration; no irritation on histopathology	15-day stability window short for shelf-life needs; ex vivo porcine, not human/in vivo	[129]
CNC Pickering O/W → polymer latex	Alkyl-grafted CNC-COOH stabilizing styrene nanoemulsions (polymer latex synthesis)	Primary experimental study	Stable Pickering nanoemulsions polymerized into nanolatexes	Polymer-latex (materials chemistry), not topical delivery—corrected placement	[33]
Bacterial cellulose (cosmetics/drug delivery review)	Breadth review: BC applications in food, cosmetics, drug delivery	Narrative review	BC used in facial masks, contact lenses, transdermal/protein delivery, tissue engineering	Feasibility survey; predates 2020s primary studies	[130]

**Table 8:** Critical appraisal of commercialization- and manufacturing-relevant studies.

System Type	Goal	Design	Key Result	Limitation/Gap	Ref.
BC reviews (non-emulsion)	Bacterial cellulose (production, modification, cosmetics/food reviews)	Survey BC production, in situ/ex situ modification, and diverse application portfolio	BC crystallinity 84–89%; water-holding >100×; documented applications in wound healing, vascular prosthetics, regenerative medicine across four reviews (2017–2020)	Reviews collectively conclude further commercial development is needed; do not cover BC in Pickering-emulsion context	[130,132,135,136]
Cellulose nanocrystals (industrial-scale; review)	Benchmarking review: CNC transition from lab to industrial production	Benchmarking/critical review	Multiple producers (CelluForce, 1-tonne/day) by mid-2010s; CNC properties vary by supplier and hydrolysis method	Supplier-to-supplier CNC variability rarely controlled for in downstream biomedical studies	[23]
Cellulose nanocrystals (patent review; non-emulsion)	Patent landscape for nanocellulose preparation, composites, applications (~2012)	Patent-landscape review	Acid/enzymatic hydrolysis, oxidation, electrospinning, homogenization, steam explosion as dominant claimed methods	Decade-old snapshot; misses 2018–2025 surge in Pickering-emulsion antimicrobial/vaccine-adjuvant patents	[134]
Cellulose nanofibrils (enzymatic pretreatment; non-emulsion)	LPMO enzymatic pretreatment as greener route to CNFs from birchwood	Primary experimental study	LPMO releases C1-oxidized oligosaccharides; subsequent shearing yields gel-like nanoscale CNFs without morphological change	Materials/process-chemistry only; not tested in emulsion, drug delivery, or antimicrobial context	[24]
PHB/cellulose-acetate-phthalate microspheres (solvent evaporation)	Colon-targeted 5-fluorouracil delivery; blend ratio optimization	Primary experimental study	29–67 μm spherical microspheres; no adverse drug–polymer interaction (FTIR/DSC); pH-responsive release supported	Colon-targeting inferred from in vitro dissolution; cytotoxicity/antitumor data are a separate follow-up study	[68]
Cellulose acetate from cigarette waste (electrospun nanofiber; non-emulsion)	Recover CA from cigarette filters; encapsulate tetracycline-HCl in CA nanofibrous membrane	Primary experimental study	Functional CA recovered and electrospun; TCH-loaded nanofibers show controlled release	Batch-to-batch variability from filter manufacturers unaddressed; residual leachables not characterized—regulatory prerequisite	[133]

**Table 9:** Regulatory and translational gaps for cellulose-emulsion products.

Gap	Why It Matters	Evidence Status	Refs.
Characterization standards	Batch consistency, review clarity	Fragmented, non-harmonized	[138,139]
Nanomaterial safety data	Systemic/occupational exposure	Emerging, incomplete	[138,139]
Dosage-form performance	Reproducible release/efficacy	In vitro heavy; in vivo scarce (partial exception: ref. [68])	[65,66,67,140]
Composite-system safety	Multifunctional carriers	Early-stage	[137,141]
Manufacturing scale-up	GMP-grade supply	Advancing (LPMO, benchmarking)	[24,34]
Patent/IP clarity	Freedom to operate	Dated landscape (needs update)	[134]

**Table 10:** Quantitative overview of the literature coverage and depth of critical analysis by application theme.

Application Theme	Primary Studies Appraised	Anchor Studies (Spotlight)	Refs.
Drug encapsulation & formulation	5 (Table 4)	Curcumin/CNC [58], Ibuprofen/CNF [60], Glipizide EC [61], Nifedipine EC/CA [66], Prednisolone CD [67]	[17]
Antimicrobial & wound care	6 (Table 5)	Oregano/CNC [76], Cinnamon/CNC [77], BC/thyme [96], Tea tree/CNF [97], Thyme threshold [75], Hemostasis [95]	[48]
Vaccine adjuvants	3 (Table 6)	Droplet-size benchmark [118], Green NPE [119], Chlamydia/IL-12 [121]	[4]
Topical & transdermal delivery	2 (Table 7)	W/O/W oligonucleotide [127], CNC-g-PIPP/bifonazole [129]	[11]
Commercialization & translation	5 (Table 8)	CNC benchmarking [23], LPMO manufacturing [24], CA waste valorization [133], CA/PHB microspheres [68], BC modification [135]	[10]
Regulatory gaps	2 (Table 9)	PVA-CMC/ZnO composite [137], Polysaccharide nanoparticle safety [138]	[11]

## Data Availability

No new data were created or analyzed in this study. Data sharing is not applicable to this article.

## Abbreviations

ZnO	Zinc Oxide Nanoparticles
XRD	X-ray Diffraction
W/O/W	Water-in-Oil-in-Water
W/O	Water-in-Oil
USP/NF	United States Pharmacopeia/National Formulary
TGA	Thermogravimetric Analysis
TAPPI	Technical Association of Pulp and Paper Industry
SEM	Scanning Electron Microscopy
PVA	Polyvinyl Alcohol
PIPP	Poly(isopropyl acrylamide-co-pyridyl disulfide methacrylate)
PHB	Poly(3-hydroxybutyrate)
O/W	Oil-in-Water
MIC	Minimum Inhibitory Concentration
MCC	Microcrystalline Cellulose
MC	Methyl Cellulose
LPMO	Lytic Polysaccharide Monooxygenase
LCST	Lower Critical Solution Temperature
ISO	International Organization for Standardization
IP	Intellectual Property
IL-12	Interleukin-12
ICH	International Council for Harmonisation
HPMC	Hydroxypropyl Methylcellulose
HP-β-CD	Hydroxypropyl-β-Cyclodextrin
HIPE	High Internal Phase Emulsion
GMP	Good Manufacturing Practice
FTIR	Fourier-Transform Infrared Spectroscopy
FDA	Food and Drug Administration
EMA	European Medicines Agency
EC	Ethyl Cellulose
DSC	Differential Scanning Calorimetry
CNFs	Cellulose Nanofibers
CNCs	Cellulose Nanocrystals
CMC	Carboxymethyl Cellulose
CAP	Cellulose Acetate Phthalate
BCS	Biopharmaceutics Classification System
BC	Bacterial Cellulose
AFM	Atomic Force Microscopy
ACS	American Chemical Society

## References

[1] Kumar V., Rastogi V. (2025). Advancements in solubility and bioavailability enhancement of BCS class II drugs: A comprehensive review using quality by design approach for self-emulsifying drug delivery systems. Orient. J. Chem..

[2] Jannin V., Chevrier S., Michenaud M., Dumont C., Belotti S., Chavant Y., Demarne F. (2015). Development of self emulsifying lipid formulations of BCS class II drugs with low to medium lipophilicity. Int. J. Pharm..

[3] Tai Z., Huang Y., Zhu Q., Wu W., Yi T., Chen Z. (2020). Utility of Pickering emulsions in improved oral drug delivery. Drug Discov. Today.

[4] Li Z., Wu H., Yang M., Xu D., Chen J., Feng H. (2018). Stability mechanism of O/W Pickering emulsions stabilized with regenerated cellulose. Carbohydr. Polym..

[5] Ahmadi S., Khormali A. (2025). Petroleum emulsion stability and separation strategies: A comprehensive review. ChemEngineering.

[6] Fujisawa S., Togawa E., Kuroda K. (2017). Nanocellulose-stabilized Pickering emulsions and their applications. Sci. Technol. Adv. Mater..

[7] Harman C.L.G., Patel M.A., Guldin S., Davies G.L. (2019). Recent developments in Pickering emulsions for biomedical applications. Curr. Opin. Colloid Interface Sci..

[8] Wu J., Ma G.H. (2016). Recent studies of Pickering emulsions: Particles make the difference. Small.

[9] Dumanli A.G. (2017). Nanocellulose and its composites for biomedical applications. Curr. Med. Chem..

[10] Marto J., Ascenso A., Simoes S., Almeida A.J. (2016). Pickering emulsions: Challenges and opportunities in topical delivery. Expert Opin. Drug Deliv..

[11] Pandey A. (2021). Pharmaceutical and biomedical applications of cellulose nanofibers: A review. Environ. Chem. Lett..

[12] Nargesi Khoramabadi H., Arefian M., Hojjati M., Tajzad I., Mokhtarzade A., Mazhar M., Jamavari A. (2020). A review of polyvinyl alcohol/carboxymethyl cellulose (PVA/CMC) composites for various applications. J. Compos. Compd..

[13] Pettignano A., Charlot A., Fleury E. (2019). Carboxyl-functionalized derivatives of carboxymethyl cellulose: Towards advanced biomedical applications. Polym. Rev..

[14] Zhang W., Liu Y., Xuan Y., Zhang S. (2022). Synthesis and applications of carboxymethyl cellulose hydrogels. Gels.

[15] Andresen M., Stenius P. (2007). Water-in-oil emulsions stabilized by hydrophobized microfibrillated cellulose. J. Dispers. Sci. Technol..

[16] Varanasi S., Henzel L., Mendoza L., Prathapan R. (2018). Pickering emulsions electrostatically stabilized by cellulose nanocrystals. Front. Chem..

[17] Zoppe J.O., Venditti R.A., Rojas O.J. (2012). Pickering emulsions stabilized by cellulose nanocrystals grafted with thermo-responsive polymer brushes. J. Colloid Interface Sci..

[18] Bizmark N., Ioannidis M.A. (2017). Ethyl cellulose nanoparticles at the alkane-water interface and the making of Pickering emulsions. Langmuir.

[19] Melzer E., Kreuter J., Daniels R. (2003). Ethylcellulose: A new type of emulsion stabilizer. Eur. J. Pharm. Biopharm..

[20] Carvalho-Guimarães F.B.D., Correa K.L., Souza T.P.D. (2022). A review of Pickering emulsions: Perspectives and applications. Pharmaceuticals.

[21] Lam S., Velikov K.P., Velev O.D. (2014). Pickering stabilization of foams and emulsions with particles of biological origin. Curr. Opin. Colloid Interface Sci..

[22] Bai L., Huan S., Xiang W., Rojas O.J. (2018). Pickering emulsions by combining cellulose nanofibrils and nanocrystals: Phase behavior and depletion stabilization. Green Chem..

[23] Reid M.S., Villalobos M., Cranston E.D. (2017). Benchmarking cellulose nanocrystals: From the laboratory to industrial production. Langmuir.

[24] Moreau C., Tapin-Lingua S., Grisel S., Gimbert I. (2019). Lytic polysaccharide monooxygenases (LPMOs) facilitate cellulose nanofibrils production. Biotechnol. Biofuels.

[25] Song M., Jiang J., Qin H., Ren X. (2020). Flexible and super thermal insulating cellulose nanofibril/emulsion composite aerogel with quasi-closed pores. ACS Appl. Mater. Interfaces.

[26] Yan H., Chen X., Song H., Li J., Feng Y., Shi Z. (2017). Synthesis of bacterial cellulose and bacterial cellulose nanocrystals for their applications in the stabilization of olive oil Pickering emulsion. Food Hydrocoll..

[27] Liu S., Jin M., Chen Y., Gao H., Shi X., Cheng W. (2017). High internal phase emulsions stabilised by supramolecular cellulose nanocrystals and their application as cell-adhesive macroporous hydrogel monoliths. J. Mater. Chem. B.

[28] Liu W., Du H., Zhang M., Liu K., Liu H., Xie H. (2020). Bacterial cellulose-based composite scaffolds for biomedical applications: A review. ACS Sustain. Chem. Eng..

[29] Hu Z., Marway H.S., Kasem H., Pelton R. (2016). Dried and redispersible cellulose nanocrystal Pickering emulsions. ACS Macro Lett..

[30] Tasset S., Cathala B., Bizot H., Capron I. (2014). Versatile cellular foams derived from CNC-stabilized Pickering emulsions. RSC Adv..

[31] Xie J., Luo Y., Chen Y., Liu Y., Ma Y., Zheng Q. (2019). Redispersible Pickering emulsion powder stabilized by nanocrystalline cellulose combining with cellulosic derivatives. Carbohydr. Polym..

[32] Jimenez-Saelices C., Seantier B. (2018). Thermal superinsulating materials made from nanofibrillated cellulose-stabilized Pickering emulsions. ACS Appl. Mater. Interfaces.

[33] Zhang Y., Karimkhani V., Makowski B.T. (2017). Nanoemulsions and nanolatexes stabilized by hydrophobically functionalized cellulose nanocrystals. Macromolecules.

[34] Vanderfleet O.M., Cranston E.D. (2021). Production routes to tailor the performance of cellulose nanocrystals. Nat. Rev. Mater..

[35] Liu W., Liu K., Wang Y., Lin Q., Liu J., Du H. (2022). Sustainable production of cellulose nanofibrils from kraft pulp for the stabilization of oil-in-water Pickering emulsions. Ind. Crops Prod..

[36] Popa L., Ghica M.V., Tudoroiu E.E., Ionescu D.G. (2022). Bacterial cellulose—A remarkable polymer as a source for biomaterials tailoring. Materials.

[37] Lahiri D., Nag M., Dutta B., Dey A., Sarkar T., Pati S., Edinur H.A., Abdul Kari Z., Mohd Noor N.H., Ray R.R. (2021). Bacterial cellulose: Production, characterization, and application as antimicrobial agent. Int. J. Mol. Sci..

[38] Khine Y.Y., Stenzel M.H. (2020). Surface modified cellulose nanomaterials: A source of non-spherical nanoparticles for drug delivery. Mater. Horiz..

[39] Tang C., Spinney S., Shi Z., Tang J., Peng B., Luo J. (2018). Amphiphilic cellulose nanocrystals for enhanced Pickering emulsion stabilization. Langmuir.

[40] Zuppolini S., Salama A., Cruz-Maya I., Guarino V. (2022). Cellulose amphiphilic materials: Chemistry, process and applications. Pharmaceutics.

[41] Jia X., Xu R., Shen W., Xie M., Abid M., Jabbar S. (2015). Stabilizing oil-in-water emulsion with amorphous cellulose. Food Hydrocoll..

[42] Gao J., Qiu Y., Chen F., Zhang L., Wei W., An X. (2023). Pomelo peel derived nanocellulose as Pickering stabilizers: Fabrication of Pickering emulsions and their potential as sustained-release delivery systems for lycopene. Food Chem..

[43] Low L.E., Tan L.T.H., Goh B.H., Tey B.T., Ong B.H. (2019). Magnetic cellulose nanocrystal stabilized Pickering emulsions for enhanced bioactive release and human colon cancer therapy. Int. J. Biol. Macromol..

[44] Ming Y., Xia Y., Ma G. (2022). Aggregating particles on the O/W interface: Tuning Pickering emulsion for the enhanced drug delivery systems. Aggregate.

[45] Yan H., Chen X., Feng M., Shi Z., Zhang W. (2019). Entrapment of bacterial cellulose nanocrystals stabilized Pickering emulsions droplets in alginate beads for hydrophobic drug delivery. Colloids Surf. B Biointerfaces.

[46] Zhang X., Wang D., Liu S., Tang J. (2022). Bacterial cellulose nanofibril-based Pickering emulsions: Recent trends and applications in the food industry. Foods.

[47] Zhang Z., Cheng M., Gabriel M.S., Neto Â.A.T. (2019). Polymeric hollow microcapsules (PHM) via cellulose nanocrystal stabilized Pickering emulsion polymerization. J. Colloid Interface Sci..

[48] Aravamudhan A., Ramos D.M., Nip J. (2013). Cellulose and collagen derived micro-nano structured scaffolds for bone tissue engineering. J. Biomed. Nanotechnol..

[49] Khalil H.P.S.A., Adnan A.S., Yahya E.B., Olaiya N.G. (2020). A review on plant cellulose nanofibre-based aerogels for biomedical applications. Polymers.

[50] Mauricio M.R., Costa P.G.D., Haraguchi S.K. (2015). Synthesis of a microhydrogel composite from cellulose nanowhiskers and starch for drug delivery. Carbohydr. Polym..

[51] Pourmadadi M., Darvishan S., Abdouss M. (2023). pH-responsive polyacrylic acid (PAA)-carboxymethyl cellulose (CMC) hydrogel incorporating halloysite nanotubes (HNT) for controlled curcumin delivery. Ind. Crops Prod..

[52] Liu H., Zhang L., Shi P., Zou Q., Zuo Y. (2010). Hydroxyapatite/polyurethane scaffold incorporated with drug-loaded ethyl cellulose microspheres for bone regeneration. J. Biomed. Mater. Res. B.

[53] Maulvi F.A., Lakdawala D.H., Shaikh A.A., Desai A.R. (2016). In vitro and in vivo evaluation of novel implantation technology in hydrogel contact lenses for controlled drug delivery. J. Control. Release.

[54] Eshaghi M.M., Pourmadadi M., Rahdar A. (2022). Novel carboxymethyl cellulose-based hydrogel with core--shell Fe3O4@sio2 nanoparticles for quercetin delivery. Materials.

[55] Chambin O., Champion D., Debray C. (2004). Effects of different cellulose derivatives on drug release mechanism studied at a preformulation stage. J. Control. Release.

[56] Meng Q., Xue Z., Chen S., Wu M., Lu P. (2023). Smart antimicrobial Pickering emulsion stabilized by pH-responsive cellulose-based nanoparticles. Int. J. Biol. Macromol..

[57] Liang Y., Zhu H., Wang L., He H., Wang S. (2020). Biocompatible smart cellulose nanofibres for sustained drug release via pH and temperature dual-responsive mechanism. Carbohydr. Polym..

[58] Ching Y.C., Gunathilake T., Chuah C.H., Ching K.Y. (2019). Curcumin/tween 20-incorporated cellulose nanoparticles with enhanced curcumin solubility for nano-drug delivery: Characterization and in vitro evaluation. Cellulose.

[59] Miao W., Gu R., Shi X., Zhang J., Yu L., Xiao H. (2024). Indicative bacterial cellulose films incorporated with curcumin-embedded Pickering emulsions: Preparation, antibacterial performance, and mechanism. Chem. Eng. J..

[60] Li Z., Yu D. (2023). Controlled ibuprofen release from Pickering emulsions stabilized by pH-responsive cellulose-based nanofibrils. Int. J. Biol. Macromol..

[61] Phutane P., Lotlikar V., Ghule A., Sutar S. (2010). In vitro evaluation of novel sustained release microspheres of glipizide prepared by the emulsion solvent diffusion-evaporation method. J. Young Pharm..

[62] Sharma M., Choudhury P.K., Jain K.L. (2017). Formulation & evaluation of ethyl cellulose microspheres containing glipizide by emulsification method. Eur. J. Biomed. Pharm. Sci..

[63] Pokharel M., Jamil M.F., Wilson J.P., Ferreira T. (2023). Sustained release of salicylic acid from ethyl cellulose microspheres fabricated using quasi-emulsion solvent diffusion method. Biomed. Eng. Adv..

[64] Alam M.S., Chowdhury J.A., Sadat S.M.A. (2015). Development and evaluation of salbutamol sulphate loaded ethyl cellulose microcapsules using emulsion solvent evaporation technique. Bangladesh Pharm. J..

[65] Irisappan S.C., Kumar B.P., Jayaveera K.N. (2013). Characterization of glibenclamide loaded cellulose acetate microparticles prepared by an emulsion solvent evaporation method. J. Pharm. Res..

[66] Soppimath K.S., Kulkarni A.R. (2001). Encapsulation of antihypertensive drugs in cellulose-based matrix microspheres: Characterization and release kinetics of microspheres and tableted microspheres. J. Microencapsul..

[67] Palanisamy M., Khanam J. (2011). Cellulose-based matrix microspheres of prednisolone inclusion complex: Preparation and characterization. AAPS PharmSciTech.

[68] Chaturvedi K., Kulkarni A.R. (2011). Blend microspheres of poly(3-hydroxybutyrate) and cellulose acetate phthalate for colon delivery of 5-fluorouracil. Ind. Eng. Chem. Res..

[69] Gupta B., Mishra V., Gharat S., Momin M., Omri A. (2021). Cellulosic polymers for enhancing drug bioavailability in ocular drug delivery systems. Pharmaceuticals.

[70] Liakos I.L., Iordache F., Carzino R., Scarpellini A. (2018). Cellulose acetate-essential oil nanocapsules with antimicrobial activity for biomedical applications. Colloids Surf. B Biointerfaces.

[71] Mikulcová V., Bordes R. (2016). On the preparation and antibacterial activity of emulsions stabilized with nanocellulose particles. Food Hydrocoll..

[72] Oun A.A., Shin G.H., Kim J.T. (2022). Multifunctional poly(vinyl alcohol) films using cellulose nanocrystals/oregano and cellulose nanocrystals/cinnamon Pickering emulsions: Effect of oil type and loading. Int. J. Biol. Macromol..

[73] Pontes-Quero G.M., Esteban-Rubio S. (2021). Oregano essential oil micro- and nanoencapsulation with bioactive properties for biotechnological and biomedical applications. Front. Bioeng. Biotechnol..

[74] Wu M., Xue Z., Wang C., Wang T., Zou D., Lu P. (2024). Smart antibacterial nanocellulose packaging film based on pH-stimulate responsive microcapsules synthesized by Pickering emulsion template. Carbohydr. Polym..

[75] Romulo A., Anjani V.S., Wardana A.A. (2024). Enhancing antimicrobial activity of thyme essential oil through cellulose nanocrystals-stabilized Pickering emulsions. Foods.

[76] Zhou Y., Sun S., Bei W., Zahi M.R., Yuan Q. (2018). Preparation and antimicrobial activity of oregano essential oil Pickering emulsion stabilized by cellulose nanocrystals. Int. J. Biol. Macromol..

[77] Liu J., Song F., Chen R., Deng G., Chao Y., Yang Z. (2022). Effect of cellulose nanocrystal-stabilized cinnamon essential oil Pickering emulsions on structure and properties of chitosan composite films. Carbohydr. Polym..

[78] Thongsrikhem N., Taokaew S., Sriariyanun M. (2022). Antibacterial activity in gelatin-bacterial cellulose composite film by thermally crosslinking with cinnamaldehyde towards food packaging application. Food Packag. Shelf Life.

[79] Pan Z., Zhong W., Xu J., Li D., Lin J., Wu W. (2024). Effects of oregano essential oil Pickering emulsion and ZnO nanoparticles on the properties and antibacterial activity of konjac glucomannan/carboxymethyl chitosan nanocomposite films. RSC Adv..

[80] Hou Y., Sun Y., Jia S., Su W., Cheng S., Tan M. (2024). Double-layer films based on konjac gum and hydroxypropyl methyl cellulose loaded with thyme essential oil Pickering emulsion: Preparation, characterization, and application. Food Hydrocoll..

[81] Li X., Ren Z., Wang R., Liu L., Zhang J., Ma F. (2021). Characterization and antibacterial activity of edible films based on carboxymethyl cellulose, Dioscorea opposita mucilage, glycerol and ZnO nanoparticles. Food Chem..

[82] Moghimi R., Aliahmadi A., Rafati H. (2017). Antibacterial hydroxypropyl methyl cellulose edible films containing nanoemulsions of Thymus daenensis essential oil for food packaging. Carbohydr. Polym..

[83] Tsai Y.H., Yang Y.N., Ho Y.C., Tsai M.L., Mi F.L. (2018). Drug release and antioxidant/antibacterial activities of silymarin-zein nanoparticle/bacterial cellulose nanofiber composite films. Carbohydr. Polym..

[84] Hosen M.I., Pranta A.D., Hasan M.M., Islam M.S., Islam T. (2024). Preparation and application of black cumin seed oil emulsion with enhanced stability for antimicrobial treatment of cellulosic fabric. Fibers Polym..

[85] Amrani M., Pourshamohammad S., Tabibiazar M. (2023). Antimicrobial activity and stability of Satureja khuzestanica essential oil Pickering emulsions stabilized by starch nanocrystals and bacterial cellulose nanofibers. Food Biosci..

[86] Farahani H., Barati A., Arjomandzadegan M. (2020). Nanofibrous cellulose acetate/gelatin wound dressing endowed with antibacterial and healing efficacy using nanoemulsion of Zataria multiflora. Int. J. Biol. Macromol..

[87] Jatoi A.W., Kim I.S., Ni Q.Q. (2019). Cellulose acetate nanofibers embedded with AgNPs anchored TiO2 nanoparticles for long term excellent antibacterial applications. Carbohydr. Polym..

[88] Peng K., Huang Y., Peng N., Chang C. (2022). Antibacterial nanocellulose membranes coated with silver nanoparticles for oil/water emulsions separation. Carbohydr. Polym..

[89] Yin N., Du R., Zhao F., Han Y., Zhou Z. (2020). Characterization of antibacterial bacterial cellulose composite membranes modified with chitosan or chitooligosaccharide. Carbohydr. Polym..

[90] Yue X., Zhang T., Yang D., Qiu F., Li Z., Wei G. (2019). Ag nanoparticles coated cellulose membrane with high infrared reflection, breathability and antibacterial property for human thermal insulation. J. Colloid Interface Sci..

[91] Zhang L., Zheng S., Hu Z., Zhong L., Wang Y., Zhang X. (2020). Preparation of polyvinyl alcohol/bacterial-cellulose-coated biochar--nanosilver antibacterial composite membranes. Appl. Sci..

[92] Horue M., Silva J.M., Berti I.R., Brandão L.R., Barud H.S. (2023). Bacterial cellulose-based materials as dressings for wound healing. Pharmaceutics.

[93] Elsherbiny D.A., Abdelgawad A.M., Shaheen T.I. (2023). Thermoresponsive nanofibers loaded with antimicrobial α-aminophosphonate-O/W emulsion supported by cellulose nanocrystals for smart wound care patches. Int. J. Biol. Macromol..

[94] Raghavendra G.M., Jayaramudu T., Varaprasad K. (2013). Cellulose--polymer--Ag nanocomposite fibers for antibacterial fabrics/skin scaffolds. Carbohydr. Polym..

[95] Ouyang X., Zhao L., Jiang F., Ling J., Yang L.Y., Wang N. (2022). Cellulose nanocrystal/calcium alginate-based porous microspheres for rapid hemostasis and wound healing. Carbohydr. Polym..

[96] Xu Y., Xin J., Lyu Y., Zhang C. (2024). Effects of bacterial cellulose/thyme essential oil emulsion coating on the shelf life of chilled chicken meat. J. Sci. Food Agric..

[97] Ferreira G.S., Silva D.J.D., Souza A.G. (2023). Eco-friendly and effective antimicrobial Melaleuca alternifolia essential oil Pickering emulsions stabilized with cellulose nanofibrils against bacteria and SARS-CoV-2. Int. J. Biol. Macromol..

[98] Wu M., Zhou Z., Yang J., Zhang M., Cai F., Lu P. (2021). ZnO nanoparticles stabilized oregano essential oil Pickering emulsion for functional cellulose nanofibrils packaging films with antimicrobial and antioxidant activity. Int. J. Biol. Macromol..

[99] Wu M., Yang J., Chen S., Lu P., Wang R. (2021). TOCNC-g-PEI nanoparticle encapsulated oregano essential oil for enhancing the antimicrobial activity of cellulose nanofibril packaging films. Carbohydr. Polym..

[100] Yang W., Zhang S., Hu Y., Fu Q., Cheng X., Li Y. (2024). Pectin-based film activated with carboxylated cellulose nanocrystals-stabilized oregano essential oil Pickering emulsion. Food Hydrocoll..

[101] Chen Q.J., You N., Liang C.Y., Xu Y.N., Wang F. (2023). Effect of cellulose nanocrystals-loaded ginger essential oil emulsions on the physicochemical properties of mung bean starch composite film. Ind. Crops Prod..

[102] Chen C., Deng S., Tian H., Yu H., Huang J., Lou X. (2025). Enhanced fresh walnut preservation using chitosan films reinforced with cinnamon essential oil and bacterial cellulose Pickering emulsion. Food Hydrocoll..

[103] Huang Y., Liu H., Liu S., Li S. (2020). Cinnamon cassia oil emulsions stabilized by chitin nanofibrils: Physicochemical properties and antibacterial activities. J. Agric. Food Chem..

[104] Li D., Li E.J., Li L., Li B., Jia S., Xie Y. (2024). Effect of TEMPO-oxidized bacterial cellulose nanofibers stabilized Pickering emulsion of cinnamon essential oil on structure and properties of gelatin composite films. Int. J. Biol. Macromol..

[105] Souza A.G., Ferreira R.R., Aguilar E.S.F., Zanata L. (2021). Cinnamon essential oil nanocellulose-based Pickering emulsions: Processing parameters effect on their formation, stabilization, and antimicrobial activity. Polysaccharides.

[106] Sun H., Li S., Chen S., Wang C., Liu D., Li X. (2020). Antibacterial and antioxidant activities of sodium starch octenylsuccinate-based Pickering emulsion films incorporated with cinnamon essential oil. Int. J. Biol. Macromol..

[107] Baldassarre F., Schiavi D., Lorenzo V.D., Biondo F. (2023). Cellulose nanocrystal-based emulsion of thyme essential oil: Preparation and characterisation as sustainable crop protection tool. Molecules.

[108] Zhou S., Peng H., Zhao A., Yang X., Lin D. (2024). Konjac glucomannan-based highly antibacterial active films loaded with thyme essential oil through bacterial cellulose nanofibers/Ag nanoparticles stabilized Pickering emulsions. Int. J. Biol. Macromol..

[109] Bu N., Huang L., Cao G., Lin H., Pang J., Wang L. (2022). Konjac glucomannan/pullulan films incorporated with cellulose nanofibrils-stabilized tea tree essential oil Pickering emulsions. Colloids Surf. A Physicochem. Eng. Asp..

[110] Yu H., Huang G., Ma Y., Liu Y., Huang X., Zheng Q. (2021). Cellulose nanocrystals based clove oil Pickering emulsion for enhanced antibacterial activity. Int. J. Biol. Macromol..

[111] Elbhnsawi N.A., Elwakil B.H., Hassanin A.H., Shehata N. (2023). Nano-chitosan/eucalyptus oil/cellulose acetate nanofibers: Manufacturing, antibacterial and wound healing activities. Membranes.

[112] Cai F., Duan Z., Yu D., Song Z., Lu P. (2025). Antimicrobial packaging activity enhancement by lemon essential oil Pickering emulsion stabilized with nanocellulose microgel particles. Food Packag. Shelf Life.

[113] He K., Sheng W., Yang L., Yang Y., Tang T., Wang C. (2024). Novel carboxymethyl cellulose/gelatin-based film incorporated with zein-stabilized lemon essential oil Pickering emulsion for the preservation of cherries. Foods.

[114] Moustafa H., Nasr H.E., Youssef A.M. (2024). Development of antibacterial carboxymethyl cellulose/quaternized starch bionanocomposites based on cinnamon essential oil nanoemulsion for wound healing applications. Biomass Convers. Biorefinery.

[115] Azadi S., Osanloo M., Zarenezhad E., Farjam M. (2023). Nano-scaled emulsion and nanogel containing Mentha pulegium essential oil: Cytotoxicity on human melanoma cells and effects on apoptosis regulator. BMC Complement. Med. Ther..

[116] Kamel R., El-Wakil N.A., Abdelkhalek A.F.A. (2021). Topical cellulose nanocrystals-stabilized nanoemulgel loaded with ciprofloxacin HCl with enhanced antibacterial activity and tissue regenerative properties. J. Drug Deliv. Sci. Technol..

[117] Lavoine N., Desloges I., Sillard C., Bras J. (2014). Controlled release and long-term antibacterial activity of chlorhexidine digluconate through the nanoporous network of microfibrillated cellulose. Cellulose.

[118] Shah R.R., Taccone M., Monaci E., Brito L.A., Bonci A., O’Hagan D.T., Amiji M.M., Seubert A. (2019). The droplet size of emulsion adjuvants has significant impact on their potency, due to differences in immune cell-recruitment and -activation. Sci. Rep..

[119] Zou Y., Li S., Ngai T., Zhang S., Ma G., Wu J. (2020). Green preparation of hydrogel particles-in-emulsions for simultaneous enhancement of humoral and cell-mediated immunity. Eng. Life Sci..

[120] Zeng Y., Zou F., Xia N., Li S. (2023). In-depth review of delivery carriers associated with vaccine adjuvants: Current status and future perspectives. Expert Rev. Vaccines.

[121] Shu M., Zhao L., Shi K., Lei W., Yang Y., Li Z. (2022). Chitosan particle stabilized Pickering emulsion/interleukin-12 adjuvant system for Pgp3 subunit vaccine elicits immune protection against genital chlamydial infection in mice. Front. Immunol..

[122] Shokri J., Adibkia K. (2013). Application of cellulose and cellulose derivatives in pharmaceutical industries. Cellulose-Medical, Pharmaceutical and Electronic Applications.

[123] Marques-Marinho F.D. (2013). Cellulose and its derivatives use in the pharmaceutical compounding practice. Cellulose-Medical, Pharmaceutical and Electronic Applications.

[124] Lavanya D., Kulkarni P.K., Dixit M., Raavi P.K. (2011). Sources of cellulose and their applications—A review. Int. J. Drug Formul. Res..

[125] Doelker E. (2013). Cellulose derivatives. Biopolymers I.

[126] Adeyeye M.C., Jain A.C., Ghorab M.K.M., Reilly W.J. (2002). Viscoelastic evaluation of topical creams containing microcrystalline cellulose/sodium carboxymethyl cellulose as stabilizer. AAPS PharmSciTech.

[127] Schmidts T., Dobler D., Schlupp P., Nissing C. (2010). Development of multiple W/O/W emulsions as dermal carrier system for oligonucleotides: Effect of additives on emulsion stability. Int. J. Pharm..

[128] Navti P.D., Pandey A., Nikam A.N., Padya B.S., Kalthur G. (2022). Ionic liquids assisted topical drug delivery for permeation enhancement: Formulation strategies, biomedical applications, and toxicological perspective. AAPS PharmSciTech.

[129] Hiranphinyophat S., Otaka A., Asaumi Y., Fujii S. (2021). Particle-stabilized oil-in-water emulsions as a platform for topical lipophilic drug delivery. Colloids Surf. B Biointerfaces.

[130] Ullah H., Santos H.A., Khan T. (2016). Applications of bacterial cellulose in food, cosmetics and drug delivery. Cellulose.

[131] Laugel C., Baillet A., Piemi M.P.Y., Marty J.P. (1998). Oil-water-oil multiple emulsions for prolonged delivery of hydrocortisone after topical application: Comparison with simple emulsions. Int. J. Pharm..

[132] Moniri M., Moghaddam A.B., Azizi S. (2017). Production and status of bacterial cellulose in biomedical engineering. Nanomaterials.

[133] Khan M.R., Liao S., Farooq A., Naeem M.A. (2023). Regeneration and modification of cellulose acetate from cigarette waste: Biomedical potential by encapsulation of tetracycline hydrochloride. Int. J. Biol. Macromol..

[134] Duran N., Lemes A.P. (2012). Review of cellulose nanocrystals patents: Preparation, composites and general applications. Recent Pat. Nanotechnol..

[135] Gorgieva S. (2020). Bacterial cellulose as a versatile platform for research and development of biomedical materials. Processes.

[136] Tayeb A.H., Amini E., Ghasemi S., Tajvidi M. (2018). Cellulose nanomaterials—Binding properties and applications: A review. Molecules.

[137] Darbasizadeh B., Fatahi Y., Feyzi-Barnaji B., Arabi M., Motasadizadeh H., Farhadnejad H., Moraffah F., Rabiee N. (2019). Crosslinked-polyvinyl alcohol-carboxymethyl cellulose/ZnO nanocomposite fibrous mats containing erythromycin (PVA-CMC/ZnO-EM): Fabrication, characterization and in-vitro release and anti-bacterial properties. Int. J. Biol. Macromol..

[138] Chen L., Yu L., Qi L., Eichhorn S.J., Isogai A. (2025). Cellulose nanocomposites by supramolecular chemistry engineering. Nat. Rev. Mater..

[139] Gericke M., Schulze P., Heinze T. (2020). Nanoparticles based on hydrophobic polysaccharide derivatives—Formation principles, characterization techniques, and biomedical applications. Macromol. Biosci..

[140] Nath B., Nath L.K., Mazumder B., Kumar P. (2010). Preparation and characterization of salbutamol sulphate loaded ethyl cellulose microspheres using water-in-oil-oil emulsion technique. Iran. J. Pharm. Res..

[141] Pourmadadi M., Shabestari S.M., Abdouss H., Amiri Z. (2024). Synthesis of pH-sensitive carboxymethyl cellulose/agarose/carbon quantum dots nanocarriers for quercetin delivery to A549 lung cancer using an emulsification method. BioNanoScience.

[142] Tohamy H.A.S., El-Sakhawy M., Abdel-Halim S.A. (2025). Antimicrobial Plectranthus amboinicus emulsions prepared with amphiphilic cellulose stearate. Euro-Mediterr. J. Environ. Integr..

